# Recapitulating the bone extracellular matrix through 3D bioprinting using various crosslinking chemistries

**DOI:** 10.3389/fbioe.2025.1506122

**Published:** 2025-06-05

**Authors:** Laurens Parmentier, Edward Vermeersch, Sandra Van Vlierberghe

**Affiliations:** Department of Organic and Macromolecular Chemistry, Faculty of sciences, Polymer Chemistry and Biomaterials Group (PBM), Centre of Macromolecular Chemistry (CMaC), Ghent University, Ghent, Belgium

**Keywords:** biophysical cues, biofabrication, bone extracellular matrix, mechanobiology, natural polymer, chain-growth crosslinking, step-growth crosslinking

## Abstract

Bioprinting allows to spatially organize cellular niches influencing mechanobiology into tissue engineered constructs thereby aiming to achieve a similar functional complexity as the various tissues present within bone. Natural polymer hydrogel matrices are favorably selected as part of many bioinks thanks to their level of mimicry with the bone osteoid matrix. More specifically, a variety of biophysical and biochemical cues targeting osteogenesis can be presented towards cells encapsulated in bioprinted constructs. This review focusses on delineating bioprinting targeting osteogenesis based on the printing approach (deposition-versus light-based bioprinting) and crosslinking chemistry utilized (chain- versus step-growth crosslinking). Moreover, the cell-biomaterial interactions at play within these constructs are addressed in line with currently established mechanobiology concepts. The delicate interplay between the presented cues from the encapsulating matrix, the used printing process and the maturity, source and concentration of the used cell type finally dictates the osteoregenerative outcome of a bioprinted construct. Given the advantages towards cell encapsulation associated with step-growth systems, there is a huge need to evaluate these systems in comparison to the heavily reported chain-growth systems (predominantly gelatin-methacryloyl or GelMA) towards the bioprinting of constructs serving osteogenesis. Moreover, multiple bioprinting strategies should be combined to tackle key challenges in the field and enable functional and scalable hierarchical constructs serving osteogenesis with incorporation of vascularization and innervation.

## 1 Introduction

Bone is the second most transplanted organ worldwide, as 5%–10% of all fractures fail to heal properly, often leading to delayed or non-union (Calori et al., 2011; Turnbull et al., 2018). Clinical gold standards suffer from various drawbacks and hence alternatives mimicking the composition and properties of the native extracellular matrix (ECM) are gaining increasing interest to enhance bone regeneration. The extra-/pericellular niche influencing mechanobiology provides the (stem) cell with a spatiotemporal presentation of biophysical and biochemical cues regulating its state. Previously, an overview of the biological cues towards osteogenesis supplied by the most common natural polymers was already described by Parmentier and Van Vlierberghe (2022). However, depending on the level of maturation within the differentiation cascade, different cues should be presented to ensure optimal osteogenic differentiation and hence, bone regeneration. This has been extensively covered in a recent review by Lewns et al. (2023). Herein, a short summary is given highlighting the main parameters at play at each specific stage. During the first cell attachment stage, the architecture of hydrogels in combination with ligand chemistry, bound peptides, growth factors and extracellular vesicles are important to allow a cell to optimally interact with the presented natural polymer (Lewns et al., 2023). The following mechanotransduction step consists of cellular sensing and integrating the perceived signals whereafter functional binding ligand sites are optimally clustered, all depending highly on the encapsulating matrix stiffness, visco-elasticity and susceptibility to degradation (Huebsch et al., 2010; Caliari and Burdick, 2016; Chaudhuri et al., 2016). Finally, the matrix remodeling stage is initiated through the cellular deposition of nascent proteins forming the pericellular matrix based on the requirement of matrix degradation and/or a dynamic micro-environment (Loebel et al., 2019).

The outlined mechanobiology concepts have been exploited in a variety of hydrogel matrices thanks to their mimicry with the bone non-mineralized ECM or osteoid deposited by osteoblasts which is comprised predominantly of hydrated collagen type I and further supplemented by other members of the collagen family (type III, V and X), bone-related glycosaminoglycan-bearing proteoglycans, glycoproteins, γ-carboxy glutamic acid proteins, proteolipids, metalloproteinases, growth factors, serum-derived proteins and cell-binding proteins (Boskey and Robey, 2013). In contrast to physically crosslinked hydrogels, covalently crosslinked systems, either applied as such or in combination with physical interactions, provide a superior network stability, suited for long-term applications such as bone tissue engineering (Echalier et al., 2019). Chemical chain-growth crosslinking builds on the continuous additive propagation of reactive groups to form oligomer kinetic chains until termination and benefits from its straightforward material handling resulting from its stability during longer time periods at physiological temperature (Van Hoorick et al., 2019). A variety of photo-crosslinking methods have been utilized in bioprinting, employing different photo-initiators, wavelengths and irradiation times (see Table 1–3). The crosslinked network has tunable properties by varying the modification degree, natural polymer concentration, photo-initiator concentration, dose and pre-crosslinking treatments (e.g. cooling, heating) which all influence the network density. The network density increases by increasing the modification degree and the natural polymer concentration due to an increased number of crosslinkable functionalities (Van Hoorick et al., 2017; Parmentier et al., 2024). By increasing the dose and/or photo-initiator concentration, the storage modulus (measure for the deformation energy stored by the sample which is completely available after the load is removed) increases due to an increased fraction of reacted functionalities resulting in a higher network density (Van Den Bulcke et al., 2000). Additionally, the photo-initiator and the used wavelengths also influence the network properties (De Moor et al., 2020). In general, by increasing the network density and thereby decreasing the mesh size, the storage modulus increases (i.e. measure of network stiffness) and both the swelling ratio and the degradation rate drop (Van Den Bulcke et al., 2000; Van Hoorick et al., 2017; Chansoria et al., 2021; Parmentier et al., 2024). Moreover, the mesh size impacts the mass transfer of nutrients and waste products, as well as cell processes including migration, differentiation and ECM production (Lin et al., 2011; Santos et al., 2012; Tytgat et al., 2019; Dogan et al., 2023).

**TABLE 1:** Bioink, cell density and printing parameters used during extrusion-based biofabrication serving osteogenesis. The reported bioink composition is the one optimized for bioprinting targeting osteogenesis.

Natural-based polymer 1	Natural-based polymer 2	Photo-initiator	Crosslinker	Other additives	Crosslinking method	Cell type	Cell concentration	Printing parameters	Ref.
5 w/v% type B GelMA DS 99%	—	2 mol% LAP (365 nm, 8 mW/cm^2^, 10 min)	—	—	Chain-growth	Human DPSCs	1 million cells/mL	Flow speed: 2.5 mm/s Printing speed 3 mm/s	Parmentier et al. (2023)
5 w/v% origin NS GelMA DS 60%	—	0.25 w/v% LAP (405 nm, NS, 40 s)	—	—	Chain-growth	Human DPSC	4 million cells/mL	Nozzle moving speed: 5 mm/s Pressure: 0.05 MPa	Wang et al. (2023b)
10 w/v% origin NS GelMA DS NS	—	0.25% LAP (405 nm, NS, 40 s)	—	—	Chain-growth	Human DPSC	4 million cells/mL	Nozzle moving speed: 5 mm/s Pressure: 0.065 MPa	Wang et al. (2024b)
10 w/v% origin NS GelMA DS 60%	—	0.25% LAP (405 nm, NS, 40 s)	—	—	Chain-growth	Human PDLSCs	4 million cells/mL	Nozzle moving speed: 5 mm/s Pressure: 0.065 MPa	Zhu et al. (2023)
10 w/v% origin NS GelMA DS NS	—	0.5 wt% LAP (405 nm, NS, 60 s)	—	5 w/w% Sr substituted xonotlite	Chain-growth	Rat BMSCs	2 million cells/mL	Printing speed: 8 mm/s Pressure: 0.15–0.20 MPa	Yu et al. (2024)
15 w/v% porcine type A GelMA DS 76%	—	0.3 v/v% Irgacure 2,959 (300–500 nm, 200 mW/cm^2^, 40 s)	—	—	Chain-growth	Murine MC3T3-E1	20 million cells/mL	NS	Irmak et al. (2019)
15 w/v% porcine type A microwave GelMA 1) DS 99% 2) DS 89%	—	0.3 v/v% Irgacure 2,959 (300–500 nm, 200 mW/cm^2^, 40 s)	—	—	Chain-growth	Murine MC3T3-E1	20 million cells/mL	NS	Irmak et al. (2019)
2.5 w/v% *Strept. Equi* MeHA DS 5%–7%	—	0.1 w/v% Irgacure 2,959 (365 nm, 3 mW/cm^2^, 10 min)	—	1 μg/mL BMP-2 in medium	Chain-growth	Human BMSCs	2 million cells/mL	NS	Poldervaart et al. (2017)
2 w/v% origin NS MeHA DS 15%	—	0.1% LAP (365 nm, 12 mW/cm^2^, during printing and 30 s post-printing)	—	—	Chain-growth	Human PDCs	15,000 spheroids/mL	Speed: 40 mm/s Pressure: 8 kPa	Sanchez et al. (2025)
3% origin NS MeGC (deacetylated degree ≥60%) DS 21%	—	12 µM riboflavin (430–485 nm,2,100 mW/cm^2^, 70 s)	—	—	Chain-growth	Human MG-63	1 million cells/mL	Printing speed: 6 mm/s Pneumatic pressure: 120 kPa	Chang et al. (2022)
2 wt% porcine bone MA-dECM DS 71%	2.54 wt% origin NS alginate (guluronic acid content 70%)	0.3 wt% Irgacure 2,959 (NS, 2.4 J/cm^2^)	1) 0.15 wt% CaCl_2_ mixed in ink 2) 10 wt% CaCl_2_ used to aerosol crosslink 3) immersion in 2 wt% CaCl_2_ after printing	—	Chain-growth	Human ASCs	5 million cells/mL	Moving speed: 10 mm/s Pneumatic pressure: 100 kPa	Lee et al. (2020)
Concentration NS origin NS GelMA DS 90%	Concentration NS rat BMA DS NS	0.25 w/v% LAP (405 nm, NS, during printing and 1 min post-printing)	—		Chain-growth	Rat BMSCs	0.05 million cells/mL BMA solution	Speed: NS Pressure: 80–120 kPa	Lu et al. (2025)
5 w/v% type A GelMA DS 85%	5 w/v% PEGDA and 1 w/v% origin NS mCMC DS NS	0.5 w/v% Irgacure 2,959 (365 nm, 10–12 mW/cm^2^, 2 min)	—	1 w/v% Needle shaped hydroxyapatite	Chain-growth	Human BMSCs	2 million cells/mL	Printing speed: 10 mm/s Pressure: NS	Das et al. (2024)
7 w/v% origin NS GelMA DS 60%	3 w/v% PEGDA	0.25 wt% visible light initiator (405 nm, NS, NS)	—	10 wt% MSN loaded with PRN, 1 μg/mL CGRP	Chain-growth	Rat BMSCs	0.5 million cells/mL	Printing speed: 10 mm/s Pressure: 0.2 MPa	Guo and He (2023)
7 wt% bovine type NS GelMA 0.62 mmol MA/g gelatin and 5 wt% bovine type NS GelMA 0.82 mmol MA/g gelatin	1 wt% origin NS MeHA DS NS	0.135 wt% LAP (365 nm, 0.54 J/cm^2^ per double layer)	—	5 wt% hydroxyapatite particles	Chain-growth	Human ASCs	5 million cells/mL	Speed: 0.5–1 mm/s Volume flow: 0.22 mm^3^/s	Wenz et al. (2017)
2.9 w/v% type B GelSH DS 67%	2.1 w/v% type B GelNB DS 91%	2 mol% LAP (365 nm, 8 mW/cm^2^, 10 min)	—	0.5 eq (with respect to number of thiols) TCEP	Thiol-ene step-growth	Human DPSCs	1 million cells/mL	Flow speed: 3.5 mm/s Printing speed 3 mm/s	Parmentier et al. (2023)
3.6 w/v% type B GelSH DS 67%	1.4 w/v% type B GelNBNB DS 169%	2 mol% LAP (365 nm, 8 mW/cm^2^, 10 min)	—	0.5 eq (with respect to number of thiols) TCEP	Thiol-ene step-growth	Human DPSCs	1 million cells/mL	Flow speed: 3.5 mm/s Printing speed 3 mm/s	Parmentier et al. (2023)
3.3 w/v% origin NS GelSH DS 50%	1.7 w/v% origin NS GelNB DS 97%	0.03% LAP (365 nm, NS, 20 s)	—	—	Thiol-ene step-growth	Human ASCs	2 million cells/mL	Flow rate: 5 μL/s Printing speed: 4 mm/s	Burchak et al. (2022)
3.75 w/v% porcine type A gelatin	3.75 w/v% brown algae ADA from (guluronic acid content 65%–70%) oxidation degree NS	—	0.1 M CaCl_2_ for 10 min	—	Schiff base step-growth	Murine ST2 and murine RAW 264.7 in 100:1 ratio	2 million cells/mL	Plotting speed: 20 mm/s Pressure: 120–150 kPa	Zehnder et al. (2017)
12 w/v% porcine type A gelatin (mixed with ADA, final concentration NS)	3 w/v% origin NS ADA (guluronic acid content 65%–70%) oxidation degree 30% (mixed with gelatin, final concentration NS)	—	Submersion in 0.1 M CaCl_2_ and 2.5 w/v% microbial transglutaminase for 10 min	Concentration NS amine-functionalized copper (Cu)-doped mesoporous bioactive glass nanoparticles	Schiff base step-growth	Mouse BMSCs	1 million cells/mL	Printing speed: 7–9 mm/s Printing pressure: 130–140 kPa	Zhu et al. (2022)
2 w/v% origin NS chitosan (85% deacetylated)	0.1 mg/mL origin NS hydroxyethyl cellulose (with glyoxal)	—	0.1 M β-glycerophosphate and concentration NS glyoxal	1.5 w/v% cellulose nanocrystals	Schiff base step-growth	Murine MC3T3-E1	5 million cells/mL	Printing speed: 2 mm/s Printing pressure: 20 kPa	Maturavongsadit et al. (2021)
3.75 w/v% porcine type A gelatin	2.5 w/v% brown algae ADA (guluronic acid content NS) oxidation degree NS	—	0.1 M CaCl_2_ and 2.5 w/v% microbial transglutaminase for 10 min	0.15% Ferulic acid	Schiff base and enzymatic step-growth	Murine MC3T3-E1	1 million cells/mL	Printing speed: 4.5 mm/s Pressure: 150 kPa	Bider et al. (2024)
5 w/v% bovine gelatin	2 w/v% fibrinogen (from bovine plasma) and 1 w/v% alginate (origin NS)	—	10 U/mL thrombin (from bovine plasma), 3 w/v% CaCl_2_ and 0.2 w/v% transglutaminase for 1 h	—	Enzymatic step-growth	Human osteoblasts	0.3 million cells/mL	NS	Pragnere et al. (2025)
8 w/v% *B. mori* SF	15 wt% porcine type A gelatin	—	Mushroom tyrosinase (500 U)	—	Enzymatic step-growth	Human TMSCs	2–5 million cells/mL	Deposition speed: 60 mm/min Pneumatic pressure: 200–250 kPa	Das et al. (2015)
5 w/v% *B. mori* SF	5 wt% porcine type A gelatin	—	Mushroom tyrosinase (1333 U/mL)	—	Enzymatic step-growth	Mouse TVA-BMSCs	10 million cells/mL	NS	Chawla et al. (2018)
5 w/v% *B. mori* SF (was mixed with gelatin, final concentration NS)	5 w/v% porcine type NS gelatin (was mixed with SF, final concentration NS)	—	Mushroom tyrosinase (287 U/mL, 20–30 min at room temperature)	2.6 mM CaCl_2_	Enzymatic step-growth	Human BMSCs	6.67 million cells/mL	Writing speed: 2 mm/s Pressure: 1 bar	Sharma et al. (2019)
5 wt% porcine type I collagen	—	—	1 mM genipin for 1 h	—	Small molecules step-growth	Human ASCs	1 million cells/mL	Nozzle speed: 10 mm/s Pneumatic pressure: 110–300 kPa	Kim et al. (2016)

GelMA, gelatin-methacryloyl; DS, degree of substitution (percentage of introduced functionalities with respect to the number of targeted functionalities); LAP, lithium phenyl-2; 4,6-trimethylbenzoylphosphinate; DPSCs, dental pulp-derived stem cells; NS, not specified; PDLSCs, periodontal ligament stem cells; BMSCs, bone marrow-derived stem cells; Irgacure 2,959, 2-hydroxy-1-(4-(hydroxyethoxy)-phenyl)-2-methyl-1-propanone; MeHA, methacrylated hyaluronic acid; BMP-2, bone morphogenetic protein-2; MeGC, methacrylated glycol chitosan; PDCs, periosteum-derived cells; Bone MA-dECM, bone methacrylated decellularized extracellular matrix; ASCs, adipose tissue-derived stem cells; BMA, bone matrix anhydride; PEGDA, poly (ethylene glycol) diacrylate; mCMC, methacrylated carboxymethyl cellulose; MSN, mesoporous silica nanoparticles; PRN, propranolol; CGRP, calcitonin gene-related peptide; MA, methacryloyl,GelSH, thiolated gelatin; GelNB, gelatin-norbornene; eq, equivalents; TCEP, Tris (2-carboxyethyl) phosphine; GelNBNB, gelatin-norbornene-norbornene; ADA, oxidized alginate; SF, silk fibroin; TMSCs, nasal inferior turbinate tissue-derived mesenchymal stromal cells; TVA-BMSCs, custom-made bone-marrow-derived mesenchymal stem cell line.

**TABLE 2:** Bioink, cell density and printing parameters used during drop-on-demand inkjet bioprinting (DoD) serving osteogenesis. The reported bioink composition is the one optimized for bioprinting targeting osteogenesis.

Printing technique	Natural-based polymer 1	Polymer 2	Photo-initiator	Crosslinker	Other additives	Crosslinking method	Cell type	Cell concentration	Printing parameters	Ref.
Thermal DoD	1.5 w/v% origin NS GelMA DS NS	10 w/v% PEGDMA	0.05 w/v% Irgacure 2,959 (long-wave, 4.5 mW/cm^2^, NS)	—	—	Chain-growth	Human BMSCs	6 million cells/mL	Droplet volume: 130 pL Heating pulse: 10 µs Firing frequency: 3,600 Hz	Gao et al. (2015a)
Piezoelectric DoD	3.3 w/v% origin NS GelSH DS 50%	1.7 w/v% origin NS GelNB DS 97%	0.03 w/v% LAP (365 nm, NS, 20 s)	—	—	Thiol-ene step-growth	Human ASCs	2 million cells/mL	Stroke velocity: 140 μm/s Stroke size: 35 µm Feed rate: 4 mm/s Dispensing frequency: 10 Hz	Burchak et al. (2022)

NS, not specified; GelMA, gelatin-methacryloyl; DS, degree of substitution with respect to number of targeted functionalities; PEGDMA, poly (ethylene glycol) dimethacrylate; Irgacure 2,959, 2-hydroxy-1-(4-(hydroxyethoxy)-phenyl)-2-methyl-1-propanone; BMSCs, bone marrow-derived stem cells; GelSH, thiolated gelatin; GelNB, gelatin-norbornene; LAP, lithium phenyl-2; 4,6-trimethylbenzoylphosphinate; ASCs, adipose tissue-derived stem cells.

**TABLE 3:** Bioink, cell density and printing parameters used during light-based biofabrication serving osteogenesis. The reported bioink composition is the one optimized for bioprinting targeting osteogenesis.

Printing technique	Natural-based polymer 1	Polymer 2	Photo-initiator	Other additives	Crosslinking method	Cell type	Cell concentration	Printing parameters	References
DLP	15 w/v% *B. Mori* SFMA DS 67.3%	—	0.2 wt% LAP	—	Chain-growth	Mouse MC3T3-E1	2 million cells/mL	405 nm, 334.36 mJ/cm^2^ per layer	Rajput et al. (2022)
DLP	1 wt% porcine GelMA DS 60%	10 wt% PVAMA DS 15%	0.2/2 mM Ru/SPS	1 wt% Ponceau 4R (photo-absorber)	Chain-growth	Human BMSCs	5 million cells/mL	Wavelength NS, 72.5 mJ/cm^2^ per layer	Lim et al. (2018)
DLP	10 w/v% ichthyic GelMA DS 90%	—	2/20 mM Ru/SPS	0.07 w/v% new coccine (photo-absorber)	Chain-growth	Equine BMSCs	10 million cells/mL	405 nm and 515 nm, 65 mJ/cm^2^ per layer	Levato et al. (2021)
DLP	10 w/v% porcine GelMA DS NS	3.33 w/v% origin NS dextran	0.5 w/v% LAP	—	Chain-growth	Rat bone MSCs	NS	405 nm, 60 mW/cm^2^ per layer, crosslinking time per layer NS	Tao et al. (2022)
DLP	10 w/v% porcine GelMA DS NS	3.33 w/v% origin NS dextran	0.33 w/v% LAP	—	Chain-growth	Rat DPSCs	—	405 nm, 600 mJ/cm^2^ per layer	Qian et al. (2023)
SLA	8 w% porcine type A GelMA DS NS	—	0.1 w% LAP	—	Chain-growth	Human aBSC or fBSC or iBSC or iBMSC or PMSC	20 million cells/mL	NS	Amler et al. (2021)
TPA	5 w/v% porcine type A GelMA DS 56%	—	0.05% LAP (photo-crosslinking at 365 nm using a dose of 3 J/cm^2^)	0.5 mM P2CK (photo-sensitizer)	Chain-growth	Human MSCs	2.5 million cells/mL	Two-photon laser wavelength of 780 nm, pulse width of <80 fs, 100 mW laser power at objective, ablation energy dose of 100 J/cm^2^	Gehre et al. (2024)
VP	5% origin NS GelMA DS 57%	—	0.05 w/v% LAP	—	Chain-growth	Human BMSCs (with and without HUVECS)	3 (0.6) million cells/mL	405 nm, dose NS	Gehlen et al. (2023)
VP	5 w/v% bovine GelMA DS 95%	—	0.075 w/v% LAP	—	Chain-growth	Human DPSCs	1 million cells/mL	405 nm, 500 mJ/cm^2^ + 365 nm, 4,800 mJ/cm^2^ post-curing	Duquesne et al. (2025)
VP	1.5 w/v% bovine GelNBNB DS 176%	3.5 w/v% bovine GelSH DS 72%	0.025 w/v% LAP	—	Step-growth	Human DPSCs	1 million cells/mL	405 nm, 184.95–205.50 mJ/cm^2^ + 365 nm, 4,800 mJ/cm^2^ post-curing	Duquesne et al. (2025)

DLP, digital light processing; SFMA, methacrylated silk fibroin; DS, degree of substitution (percentage of introduced functionalities with respect to the number of targeted functionalities); LAP, lithium phenyl-2; 4,6-trimethylbenzoylphosphinate; GelMA, gelatin-methacryloyl; PVAMA, methacrylated poly (vinyl alcohol); Ru, tris-bipyridylruthenium (II) hexahydrate; SPS, sodium persulfate; BMSCs, bone marrow-derived stem cells; NS, not specified; MSCs, mesenchymal stem cells (tissue type not specified); DPSCs, dental pulp-derived stem cells; SLA, stereolithography; aBSC, alveolar bone stem cells; fBSC, fibula bone stem cells; iBSC, iliac crest bone stem cells; iBMSC, iliac crest bone marrow stem cells; PMSC, mastoid periosteum stem cells; TPA, two-photon ablation; P2CK, 3,3′-((((1E,1′E)-(2-oxocyclopentane-1; 3-diylidene)-bis(methaneylylidene))-bis(4,1-phenylene))-bis(methyl-azanediyl))-dipropionate; VP, tomographic volumetric printing; HUVECs, human umbilical vein endothelial cells.

However, chain-growth crosslinked networks are characterized by inferior control of the reacted functionalities, a more heterogeneous network leading to shrinkage during crosslinking and oxygen inhibition requesting higher photo-initiator concentrations in combination with higher spatiotemporal energy which is detrimental for encapsulated cells (Van Hoorick et al., 2019; An et al., 2023). Conversely, a more homogeneous network can be presented towards encapsulated cells through the use of step-growth crosslinking mechanisms in which complementary reactive groups can only react with one another in an orthogonal reaction (Van Hoorick et al., 2019). Distinctively different properties can be presented to the encapsulated cells through modification of the step-growth network density by changing the modification degree, natural polymer/crosslinker (/photo-initiator) concentration and their applied reciprocal ratio, pre- and post-crosslinking treatments in correspondence with the chain-growth crosslinked networks. In general, by increasing the network density and thereby decreasing the mesh size, the storage modulus increases and both the swelling ratio and the degradation rate drop (Greene and Lin, 2015; Tytgat et al., 2019; Göckler et al., 2021; Van Hoorick et al., 2021; Parmentier et al., 2023; 2024). Only the chain- and step-growth crosslinking approaches which have been exploited in bioinks serving osteogenesis are further highlighted herein. For a more extensive overview covering various modifications on natural polymers that subsequently undergo chain- or step-growth crosslinking, the reader is referred to other excellent reviews (Pei et al., 2019; Sorushanova et al., 2019; Van Hoorick et al., 2019; Farokhi et al., 2021; An et al., 2023; Tan et al., 2023). As a first step-growth system, photo-crosslinkable thiol-ene systems are discussed since this crosslinking method is not susceptible to oxygen inhibition leading to lower radical concentrations while it allows faster reaction rates and higher network conversions (Bertlein et al., 2017). Nevertheless, cross-reactivity with other thiols leading to reduced stability limits the widespread application of this type of inks (Van Hoorick et al., 2019). Alternatively, Schiff base crosslinking exploits imines as reversible and dynamic crosslinks under mild and straightforward reaction conditions without the production of radical species, yet they suffer from a lack of spatiotemporal control (Echalier et al., 2019; Van Hoorick et al., 2019; Gao et al., 2021). Subsequently, enzymatic crosslinking exploits a mild crosslinking process with high selectivity and efficiency but does not allow for spatiotemporal crosslinking control (Echalier et al., 2019; Van Hoorick et al., 2019). Finally, small molecule crosslinkers have also been used to aid in the crosslinking of natural polymers yet again lack the spatiotemporal crosslinking control.

In order to incorporate the studied cellular niches influencing mechanobiology into a bio-engineered construct, bioprinting has emerged since it comprises a variety of deposition- and light-based techniques exhibiting a range of resolutions and printing speeds thereby assembling and patterning bioinks with a prescribed organization through the use of computer-aided transfer processes (Guillemot et al., 2010). Thanks to the achieved structural and compositional organization in bioprinting delivering spatial control of cell-cell and cell-ECM interactions, this active field of research enables to accommodate better for the metabolic demands of embedded cells through the use of adapted architectural designs (Malda et al., 2013; Moroni et al., 2018b). However, targeting functional complexity of the bioprinted constructs is a topic of current investigations with a specific need for defined micro-environments that mimic native tissue complexity (Malda et al., 2013; Harley et al., 2021). Therefore, to enable a profound understanding of the interactions at play to mimic the osteoid and induce osteogenic differentiation, a major process during intramembranous and a late-stage process during endochondral ossification, this review focuses on the osteoregenerative outcome of encapsulated cells in response to step- or chain-growth crosslinked natural (interpenetrating) polymer networks forming the major part of the bioink. Hence, the literature search was designed so that studies were only included that focused on cell encapsulation within a natural polymer matrix for 3D bioprinting facilitating (qualifiable/quantifiable) osteogenesis. The review is structured so that first a division is made based on the printing technique used whereafter a subdivision is made based on crosslinking chemistry employed for natural polymers. The first part of this review covers the influence of various crosslinking chemistries employed in deposition-based bioprinting incorporating both filament- and droplet-based techniques towards osteogenesis. The second part of this review entails the influence of various crosslinking chemistries applied in light-based bioprinting towards osteogenesis. A final part covers the limitations of current bioprinted hydrogel scaffolds together with recommendations for future work.

## 2 Deposition-based bioprinting of constructs targeting osteogenesis

Direct ink writing refers to all fabrication techniques using a computer-controlled translation stage, moving a pattern generating device to deposit an ink in a controlled architecture (Lewis and Gratson, 2004; Lewis, 2006). Those techniques can be subdivided into filament-based and droplet-based techniques (Lewis and Gratson, 2004; Lewis, 2006). The techniques relevant for biofabrication targeting osteogenesis are extrusion-based bioprinting, also known as 3D plotting, bioplotting or robotic dispensing, and drop-on-demand inkjet printing respectively (Table 1, 2) (Jungst et al., 2016; Moroni et al., 2018a). In this review, extrusion-based bioprinting as well as thermal and piezoelectric drop-on-demand inkjet bioprinting will be discussed extensively.

### 2.1 Extrusion-based bioprinting of constructs targeting osteogenesis

In extrusion-based bioprinting, a mechanical or pneumatic fluid dispensing system is used to force the bioink through the nozzle, resulting in a continuous filament (Cui et al., 2010; Jungst et al., 2016; Moroni et al., 2018a). The computer controls the 3D movement of the printhead in order to print in a layer-by-layer fashion according to the CAD files on a stationary printbed (Cui et al., 2010; Jungst et al., 2016). In mechanical-driven systems, a screw or piston applies the driving force allowing precise control of the extruded volume (Malda et al., 2013; Ozbolat and Hospodiuk, 2016; Wenger et al., 2022). In the former case, rotational mechanical forces are directly applied on the ink by a screw connected to the motor (Gu et al., 2020). In the latter case, the ink is extruded by linear mechanical forces exerted by the piston connected via a guide screw to the motor (Gu et al., 2020). The pneumatic-driven system applies compressed air (5–800 kPa) on the bioink (Cui et al., 2010). This approach has less control of the extruded volume as it depends on the applied pressure as well as on the rheological properties of the ink and the printing set-up (Jungst et al., 2016). Sterilization of the air via a filter is required when the air is directly applied onto the cell-laden ink (Gu et al., 2020).

During the printing process, the cells experience shear, compressive and extensional forces reducing the cell viability (80%–90%) (Chang et al., 2008; Hölzl et al., 2016; Ning et al., 2020; Xu H. et al., 2022). The forces exerted on cells in the pneumatic dispensing system are similar to those in the piston dispensing system (Ning et al., 2020). In both groups, the cells experience shear stress in the nozzle and extensional stress at regions from the needle cartridge to the needle tip (Ning et al., 2020). The screw-based system exerts additional shear stress on the encapsulated cells due to the direct ink-screw contact (Ning et al., 2020). The shear/extensional force is the dominant force causing cell damage and cell death (Paxton et al., 2017; Cidonio et al., 2019b; Boularaoui et al., 2020; Ning et al., 2020). The shear stress can be modified by changing the nozzle diameter/length, nozzle shape, printing pressure, print head speed and ink viscosity (Billiet et al., 2014; Boularaoui et al., 2020; Ning et al., 2020; Schwab et al., 2020). Ning et al. concluded that the screw-based system induces greater cell damage than the pneumatic/piston-based system making the former less suitable for biofabrication (Ning et al., 2020). Despite the risk of cell damage and cell death, shear stress within a specific range (and other mechanical forces) are biophysical cues inducing the differentiation of stem cells into specific lineages (Moehlenbrock et al., 2006; White and Frangos, 2007; Zhao et al., 2007; Dong et al., 2009; Wong et al., 2012; Boularaoui et al., 2020). When bone marrow-derived stem cells (BMSCs) are exposed to fluid flow induced-shear stress, osteogenic differentiation is induced (Yourek et al., 2010). In contrast, Blaeser et al. reported an unaltered mesenchymal stem cell phenotype during microvalve-based bioprinting upon exposure to shear stress below 15–20 kPa (Blaeser et al., 2016). Therefore, additional research is needed to determine the impact of extrusion-based bioprinting on the stem cell phenotype.

Extrusion-based technologies are promising for biofabrication. Similar to drop-on-demand inkjet printing (DoD), multiple nozzles and different inks can be combined into a heterocellular, multi-material construct. A broad range of biomaterials are compatible with extrusion-based bioprinting having a viscosity window ranging from 30 mPa.s up to 6 × 10^7^ mPa.s (Chang et al., 2011). Even higher viscosities are compatible with the printing process when a mechanical dispensing system is used (Habib et al., 2018). The used hydrogels regularly exhibit shear thinning behavior, resulting in a decreasing viscosity with increasing shear rate. Hence, when a pressure is applied during printing, the viscosity drops, allowing a smooth extrusion. Upon deposition, the shear rates drop drastically, resulting in an increasing viscosity and the preservation of the extruded shape (Chimene et al., 2016; Boularaoui et al., 2020). Extrusion-based bioprinting can be applied with bioinks encapsulating high (single) cell densities (∼10^8^ cells/mL) and spheroids, allowing printing of physiological cell densities in a hydrogel scaffold (Murphy and Atala, 2014; Diamantides et al., 2019; De Moor et al., 2021; Shao et al., 2021). Additionally, the speed can range from 2 up to 60 mm/s depending on the used system (Tarassoli et al., 2021).

Challenges associated with extrusion-based bioprinting are related to sedimentation, clogging, lack of reproducibility and (relatively) low resolution. Sedimentation of the encapsulated cells influenced by the ink’s viscosity, the density of cells and the cell-adhesion site distribution results in an inhomogeneous cell distribution (Chen et al., 2019). This is specifically valid when employing low viscosity inks and large printing times. Additionally, the low viscosity results in poor mechanical strength, hence, collapse of a multi-layered structure (Yin et al., 2018; Chen et al., 2019). Conversely, a too high viscosity results in high shear stresses, inducing cell damage and cell death. Hence, the viscosity should be carefully tuned to prevent both sedimentation and cell death/damage. Secondly, clogging caused by the accumulation of cells, particles or solidified material obstructs the ink flow through the nozzle (Shao et al., 2021). A third limitation is the sensitivity of the printing process/parameters to environmental parameters including temperature and humidity as well as batch-to-batch variability (Wenger et al., 2022). While the environmental variations can be excluded by printing in a temperature-humidity controlled room, the batch-to-batch variability requires the identification of working windows of the printing parameters including pressure, nozzle/printbed temperature, print-speed, and layer height, amongst others. Finally, the general resolution is low as compared to other biofabrication technologies (200–1,000 µm) (Hölzl et al., 2016).

#### 2.1.1 Chain-growth crosslinking

##### 2.1.1.1 Gelatin-methacryloyl with/without additives

Prior to biofabrication, an optimization must be performed to determine the network influencing variables to ensure optimal osteogenic differentiation post-printing. Researchers performed an evaluation using casted 5, 10 and 15 w/v% GelMA (DS 56%, 0.5 w/v% lithium phenyl-2,4,6-trimethylbenzoylphosphinate (LAP), 5 min irradiation with 7 mW/cm^2^ at 405 nm) encapsulating 2 million cells/mL immortalized human adipose tissue-derived stromal cells (Martinez-Garcia et al., 2021; Garcia et al., 2022). Firstly, although all concentrations exhibited similar stress relaxation (time-dependent stress reduction in response to a constant strain, around 8%), the stress relaxation of 5 w/v% GelMA increased over time and was maximal (11%) after 14 days of culture. They concluded that the hydrogel’s stress relaxation might modulate matrix metalloproteinase (MMP) expression and activation, which facilitates proteolytic matrix remodeling and cell spreading (Lutolf et al., 2003; Martinez-Garcia et al., 2021; 2022). Secondly, 5 w/v% GelMA was the only concentration resulting in active MMPs after 14 days. Moreover, the cells exhibited the highest degree of spreading and maintained their viability in 5 w/v% GelMA after 14 days (Martinez-Garcia et al., 2022). This material behavior and cell response are favorable since osteogenic differentiation is strongly correlated to the ligand-RGD (Arg-Gly-Asp) clustering obtained through local proteolytic matrix degradation along with an adequate viscoelasticity (i.e. stress relaxation time around 1 min) (Huebsch et al., 2010; Khetan et al., 2013; Chaudhuri et al., 2016). Overall, those experiments revealed the potential of 5 w/v% GelMA in bioinks targeting osteogenesis.

Despite the expression of active MMPs and maximal stress-relaxation, the use of low concentration GelMA (5 w/v%) in extrusion-based biofabrication is limited due to its low viscosity, limited temperature processing window and slow gelation rate after printing (Billiet et al., 2014; Yin et al., 2018; Cidonio et al., 2019a). Parmentier et al. evaluated the potential of extrusion bioprinted 5 w/v% GelMA scaffolds encapsulating 1 million human dental pulp stem cells (DPSCs) per mL towards osteogenesis (Parmentier et al., 2023). Prior to printing, the ink was cooled in the fridge (10 min) to increase the viscosity, hence, to facilitate printing. The obtained strut sizes and pore sizes matched with the target values confirming the printability and computer-aided design/computer-aided manufacturing (CAD-CAM) mimicry (Parmentier et al., 2023). Post-printing, the physically crosslinked construct lost its integrity during chemical crosslinking, attributed to the heat generated by ultraviolet (UV)-lamps, lowering the final pore size (Parmentier et al., 2023). A compressive modulus below the range identified for optimal osteogenesis of encapsulated (non-printed) stem cells (11–30 kPa) was obtained, (potentially) causing a too compliant matrix, thereby impairing the binding between cell-adhesive motives and integrins (Huebsch et al., 2010). Note that also a too stiff matrix is unfavorable, since cells need to deform the matrix to cluster the RGD sequences. Since this clustering is closely related to osteogenic differentiation, a lower expression of osteogenic markers is expected (and validated) with respect to compressive moduli in the range targeting osteogenesis. In general, a higher compressive modulus can be obtained by increasing the photo-crosslinkable polymer concentration, as well as the dose and photo-initiator concentration, while still obeying the cytotoxicity limit of UV-A irradiation (5.25 J/cm^2^) and LAP (1.12 mM), or by using different crosslinking strategies (Van Den Bulcke et al., 2000; Markovic et al., 2015; Wong et al., 2015; Parmentier et al., 2023). Notably, the extrusion printing process had no significant effect on the osteogenic differentiation of DPSCs evidenced by alkaline phosphatase (ALP) expression (day 7) and calcium deposition (day 28) (Wang W. et al., 2023).

**FIGURE 1:**
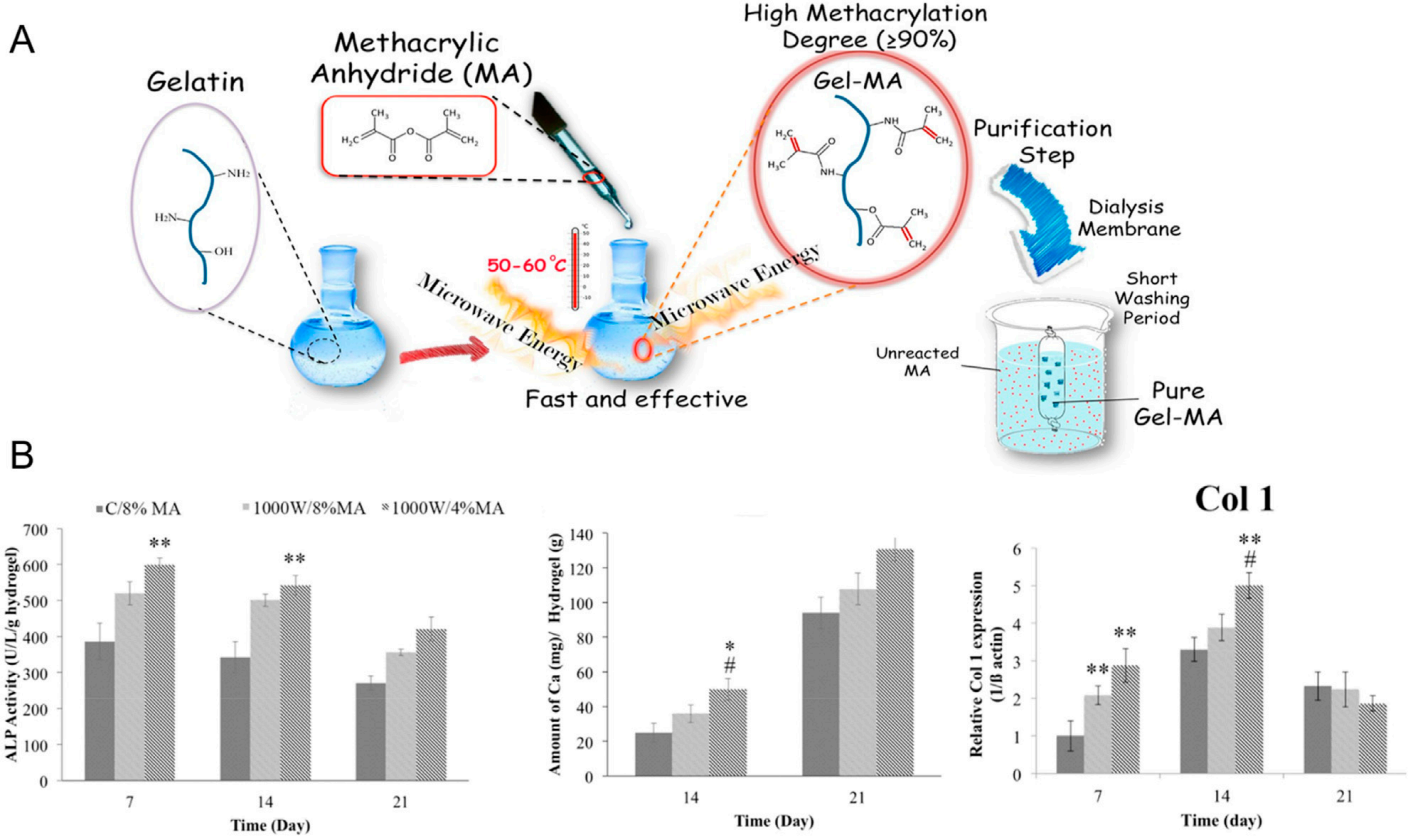
**(A)** Overview of conventional and microwave-assisted gelatin-methacryloyl (GelMA) modifications to introduce photo-crosslinkable methacryloyl moieties onto the gelatin backbone **(B)** Alkaline phosphatase (ALP) activity [U/L/g hydrogel], amount of deposited calcium [mg/g hydrogel] and relative Collagen type 1 (Col 1) expression of pre-osteoblasts encapsulated in GelMA made according to the conventional protocol using 8 v/v% methacrylic anhydride (C/8%MA) or according to the microwave method with 4 v/v% (1000W/4%MA) or 8 v/v% methacrylic anhydride (1000W/8%MA). Reproduced from Irmak et al. (2019) with permission.

Various studies have evaluated the effects of GelMA concentration, cell type and cell concentration on osteogenic differentiation following bioprinting to identify the optimal bioink formulation. Firstly, researchers assessed the osteogenic differentiation of extrusion bioprinted human DPSCs and human periodontal ligament stem cells (PDLSCs) (4 million cells/mL) in 3, 5 and 10 w/v% GelMA (DS not specified) (Zhu et al., 2023; Wang W. et al., 2024). By increasing the GelMA concentration, the compressive modulus and degradation time increased whereas the swelling ratio dropped. This can be explained by the lower mesh size upon increasing concentration. Moreover, they reported an enhanced osteogenic differentiation of either DPSCs or PDLSCs in 10 w/v% GelMA compared to 3 or 5 w/v% based on alizarin red staining (ARS) (day 21, only performed for DPSCs) and the expression of ALP, bone-morphogenetic protein-2 (BMP-2), Runt-related transcription factor 2 (RUNX2) and specificity protein-7 (SP7) (days 4, 7 and 14) (Zhu et al., 2023; Wang W. et al., 2024). Additionally, the bioprinted constructs of 10 w/v% GelMA encapsulating DPSCs were implanted into cranial defects in mice revealing nearly complete closure with new bone after 12 weeks (Wang W. et al., 2024). This discrepancy in promising GelMA concentrations (5 vs. 10 w/v%) can be attributed to the different biomaterial properties (i.e. origin, modification procedure and modification degree), applied crosslinking strategies (i.e. type and concentration of photo-initiator, gel/sol state of hydrogel pre-crosslinking, irradiation intensity, irradiation time and irradiation wavelength), protocols for measuring biophysical cues, cell formulation (i.e. type and concentration of cells) and culture conditions (i.e. composition of medium). The study of Irmark et al. exemplifies that the GelMA modification procedure influences the final biophysical properties. They extrusion bioprinted 15 w/v% GelMA (comparing different DS values) encapsulating mouse pre-osteoblasts (MC3T3-E1, 20 million cells/mL) (Irmak et al., 2019). GelMA was prepared using both the original protocol and their novel method exploiting microwaves (Figure 1A). The latter protocol enabled to reduce the reaction time and to obtain a higher DS when using equal amounts of methacrylic anhydride. Hence, the microwave-assisted modification resulted in a denser crosslinked network, thereby imparting enhanced mechanical strength. Here, the GelMA ink modified with 4 v/v% methacrylic anhydride and 1000 W microwaves (1000W/4%MA) exhibited the highest compressive modulus (60 kPa), highest storage modulus (41 kPa) and the lowest degradation rate (27% after 35 days). 1000W/4%MA also demonstrated the most pronounced effect on osteogenesis as evidenced by the highest collagen type I (COL1) expression (at day 14), ALP activity (at days 7 and 14) and calcium deposition (at days 14 and 21) (Figure 1B). An in-depth analysis of the microwaves’ impact on the gelatin backbone, the 3D polymer network after crosslinking and the resulting cellular interaction is needed to understand the obtained *in vitro* results since previous reports revealed the need of sufficient degradation and an intermediate compressive modulus to stimulate osteogenic differentiation (*vide supra*) (Huebsch et al., 2010; Khetan et al., 2013; Chaudhuri et al., 2016). The *in vitro* results might be (partially) explained by the rather high cell density applied (20 million cells/mL), as compared to other reported bioinks targeting osteogenesis, which accelerates mineralization, increases the mineral density and results in a more spread cell morphology, as well as the used cell type (Zhang et al., 2020; de Leeuw et al., 2024). Finally, a separate study reported on the optimization of the cell density within 10 w/v% GelMA (DS not specified) bioprinted constructs by evaluating the cell viability (at days 4 and 7) and ALP expression (at days 4 and 7) (Yu et al., 2024). The results demonstrated that a cell density of 2 million BMSCs per mL outperformed 0.5, 1.0 and 1.5 million BMSCs per mL (Yu et al., 2024). It would be interesting to compare the ALP activity and calcium deposition in all articles reporting pure GelMA bioinks serving osteogenesis yet having completely different mechanical properties. However, a comparison over the different studies is not possible due to the lacking standardization and uniformity (e.g. Ca deposition: mg/g hydrogel vs. ng/ng DNA). Therefore, it remains inconclusive whether one approach holds greater promise towards facilitating osteogenesis.

##### 2.1.1.2 Other methacrylated natural polymers with/without gelatin-methacryloyl

**FIGURE 2:**
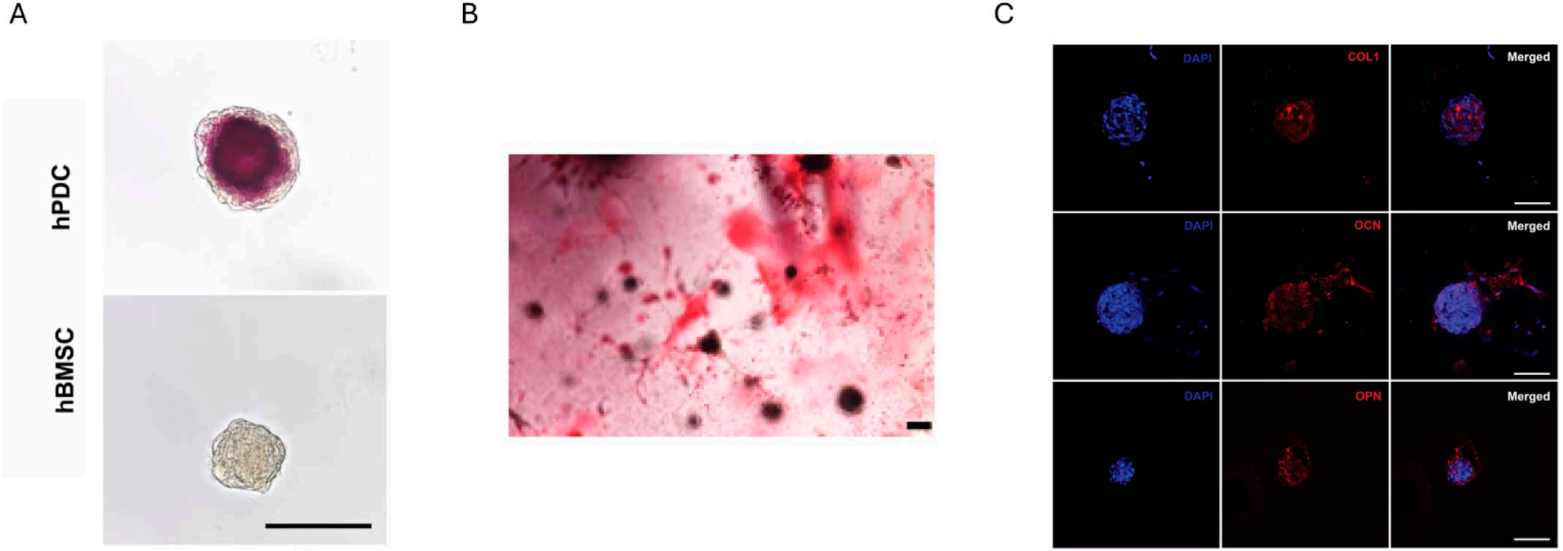
*In vitro* evaluation of methacrylated hyaluronic acid (MeHA) at day 28. **(A)** Alizarin red staining of either human periosteum-derived cell (hPDC) spheroids or human bone marrow-derived stem cell (BMSC) spheroids within moulded MeHA. **(B)** Alizarin red S staining of hPDC spheroids within extrusion bioprinted MeHA. **(C)** Immunofluorescence staining of hPDC spheroids within extrusion bioprinted MeHA for collagen type I (COL1), osteocalcin (OCN) and osteopontin (OPN). Nuclei are stained blue (DAPI). Scale bars for all images are 100 µm. Reproduced from Sanchez et al. (2025) under open access license.

Besides GelMA, also other methacrylated natural polymers were extrusion bioprinted with/without additives and photo-crosslinked post-printing. The employed polymers relate to the ECM composition being proteins (e.g. collagen), polysaccharides (e.g. hyaluronic acid) or a mixture (e.g. decellularized ECM). A first important bioink type involves stem cells encapsulated within a single methacrylated natural polymer. Hyaluronic acid is a frequently used polysaccharide due to its biodegradability, biocompatibility, and its abundance as glycosaminoglycan in the ECM. Upon esterification of its hydroxyl groups with methacrylic anhydride, methacrylated hyaluronic acid (MeHA) is obtained which has tunable mechanical properties and intrinsic osteogenicity (Poldervaart et al., 2017). Poldervaart et al. compared moulded MeHA (DS 5%–7%) encapsulating human BMSCs (2 million cells/mL) at varying concentrations (1.0, 1.5, 2.0, 2.5 and 3.0 w/v%) (Poldervaart et al., 2017). They observed a higher stiffness, lower swelling and slower degradation upon increasing MeHA concentration as well as higher calcium deposition when no additional osteogenic stimulation was added. The intrinsic osteogenicity makes MeHA an interesting candidate biomaterial in bone tissue engineering. To further exploit the use of MeHA, Sanchez et al. evaluated 2 w/v% MeHA (DS 15%) encapsulating BMSC and periosteum derived cell (PDSC) spheroids (Sanchez et al., 2025). PDSC spheroids showed a superior *in vitro* response, since only the latter spheroids resulted in a positive ARS staining (day 28) and RUNX2 expression (day 14) (Figure 2A). Additionally, the PDSC spheroids had an appropriate size (∼150 µm) to avoid a necrotic core along with significant shear stresses during extrusion bioprinting. After bioprinting, the positive ARS staining as well as the expression of COL1, osteocalcin (OCN) and osteopontin (OPN) proved the successful osteogenic differentiation of the encapsulated PDSC spheroids (Figures 2B,C). Besides hyaluronic acid, also other polysaccharides such as chitosan were investigated. Researchers selected glycol chitosan (GC) to be methacrylated because of its solubility at cell culture pH (Chang et al., 2022). Based on printability, 3% methacrylated GC (DS 21%) was selected (Chang et al., 2022). Instead of varying the polymer concentration to change the biophysical cues, the irradiation time was increased leading to an increased compressive modulus and degradation time, while the swelling ratio decreased. The MG-63 cell-laden bioprinted scaffold crosslinked at 430–485 nm with an intensity of 2,100 mW/cm^2^ for 70 s resulted in a compressive modulus within the range targeting osteogenesis and gave rise to the most pronounced ALP activity (day 4 and 7) and calcium deposition (day 4 and 7). Remarkably, it was reported by other researchers that biophysical cues (i.e. compressive modulus) resulting from a non-cell-mediated degradable, covalently crosslinked, cell-interactive hydrogel, that was not printed, showed little influence on the stem cell fate (Khetan et al., 2013). More specifically, RGD-modified MeHA did not result in osteogenic differentiation when encapsulating human mesenchymal stem cells (MSCs) in resins with compressive moduli varying from 4–92 kPa. Additionally, the introduction of proteolytically degradable crosslinks in the absence of crosslinked methacrylates facilitated osteogenic differentiation. Hence, although similar stiffnesses were obtained with similar resins, remarkably different differentiation outcomes were obtained. An explanation for this discrepancy might be the difference in hydrogel composition (e.g. polymer molecular weight, methacrylation degree, conversion) and/or cell culture parameters (e.g. cell type and culture conditions).

The ECM composition was accurately mimicked by combining methacrylated decellularized bone ECM (MA-dECM, 2 wt%, DS 71%) with alginate (2.54 wt%) and CaCl_2_ (0.15 wt%) encapsulating human adipose tissue-derived stem cells (ASCs) (5 million cells/mL) (Lee et al., 2020). The formulation was set based on rheological properties and cell viability. Interestingly, the presence of collagen, laminin, fibronectin and glycosaminoglycans was confirmed after demineralization, decellularization and methacrylation. During printing, the construct was aerosol crosslinked with 10 wt% CaCl_2_ and after printing, the construct was both ionically (in bath of 2 wt% CaCl_2_) and UV-crosslinked (0.3 wt% 2-hydroxy-1-(4-(hydroxyethoxy)-phenyl)-2-methyl-1-propanone (Irgacure 2,959), 2.4 J/cm^2^). The ALP activity analysis (day 7), ARS staining (day 7 and 14) and quantitative reverse transcription polymerase chain reaction (RT-qPCR) (day 14) showed increased osteogenesis as compared to an alginate ink (3.5 wt%, 0.5 wt% CaCl_2_ at 7:3 ratio) without MA-dECM. Given alginate’s polysaccharide nature, it lacks cell-adhesive motifs (e.g. RGD) and MMP-degradable sequences (Lee and Mooney, 2012). Therefore, the lower osteogenic differentiation observed within purely crosslinked alginate is unsurprising (*vide supra*). In literature, RGD and MMP-sensitive peptides have been grafted onto alginate to obtain a cell-interactive and biodegradable biomaterial (Lee et al., 2008; Fonseca et al., 2011).

A second important bioink formulation entails stem cells encapsulated within biopolymer blends such as GelMA combined with other (meth-) acrylated biopolymers. Such systems are generally exploited to tailor the mechanical and rheological properties of a single constituent resin. For example, low concentration GelMA has favorable properties for cell encapsulation but also demonstrates a limited extrusion printability. In alignment with the concepts discussed earlier, the mesh size drops upon increasing the concentration of additional methacrylated polymers resulting in an increased compressive modulus and degradation time as well as a lower swelling ratio (Guo and He, 2023; Das et al., 2024; Lu et al., 2025). Several examples that were combined with GelMA include MeHA, photo-crosslinkable bone matrix anhydride (BMA), methacrylated carboxymethyl cellulose (mCMC) and poly (ethylene glycol) diacrylate (PEGDA) (Wenz et al., 2017; Guo and He, 2023; Das et al., 2024; Lu et al., 2025). Hence, besides introducing RGD moieties and MMP-cleavable crosslinks onto the backbone of polysaccharides, cell interactivity can also be increased through the addition of GelMA. Wenz et al. extrusion bioprinted a bioink containing GelMA (7 wt% 0.62 mmol methacrylate functionalities/g gelatin and 5 wt% 0.82 mmol methacrylate functionalities/g gelatin), MeHA (1 wt%, DS not specified), hydroxyapatite particles (5 wt%, 12 µm) and human ASCs (5 million cells/mL) and evaluated the influence of the added particles (Wenz et al., 2017). GelMA with a higher and lower methacrylation degree were blended to tailor the hydrogel properties regarding the print-process (i.e. viscosity) and cell-encapsulation (i.e. stiffness and swelling). By increasing (decreasing) the modification degree, the intermolecular forces and triple helix formation are partially reduced (enhanced) resulting in lower (higher) viscosity and higher (lower) mechanical properties post-printing (Hoch et al., 2013). Additionally, the hydroxyapatite particles resulted in an increased storage and loss modulus, although the gelation temperature remained unaltered. Similar as before, both the hydroxyapatite particles and the differentiation medium induced osteogenic differentiation as confirmed by collagen type I and fibronectin production (day 28) as well as ALP and OPN expression (day 14). In a follow-up study, photochemically inert groups were introduced onto GelMA allowing a further reduction in viscosity while preventing an increased storage modulus post-crosslinking (Leucht et al., 2020). Hence, a toolbox of gelatins can be used to tailor the bioink properties.

#### 2.1.2 Step-growth crosslinking

##### 2.1.2.1 Thiol-ene

An often-reported alternative strategy for chain-growth crosslinking encompasses thiol-ene step-growth crosslinking. Although a variety of thiol crosslinkers (e.g. thiolated gelatin (GelSH), dithiothreitol (DTT), poly (ethylene glycol)-tetra-thiol (PEG4SH)) and alkene functionalized natural polymers (e.g. gelatin-norbornene (GelNB), hyaluronic acid-norbornene (NorHA), allyl-functionalized gelatin (GelAGE)), have been reported, only the gelatin-based thiol-norbornene system GelNB/GelSH and GelNB/PEG4SH have been used for deposition-based biofabrication serving osteogenesis. GelSH is a promising thiol crosslinker due to its biocompatibility, cell-interactivity, biodegradability and absence of phase separation, which is different for synthetic or polysaccharide backbones including PEG4SH (Greene and Lin, 2015; Shih et al., 2016; Van Nieuwenhove et al., 2016; Van Hoorick et al., 2021). Upon UV-exposure, step-growth crosslinking is initiated resulting in a homogeneous network, which is completely biodegradable and stable under culture conditions (Van Hoorick et al., 2018; 2021). Nevertheless, its use in biofabrication is challenged due to uncontrolled disulfide bond formation, leading to a viscosity increase over time (Carpentier et al., 2024). Therefore, reductants such as tris(2-carboxyethyl) phosphine (TCEP) have been added (Carpentier et al., 2024).

GelSH as multivalent thiol-crosslinker and alkene-functionalized natural polymers, including GelNB and gelatin-norbornene-norbornene (GelNBNB), have already been combined into thiol-ene bioinks serving osteogenesis. The latter polymer was obtained by modifying both primary amines and carboxylic acids with 5-norbornene-2-carboxylic acid (Parmentier et al., 2024). The thiol-ene ratio is an additional variable with respect to chain-growth system to tune the network density. While a ratio equal to unity results in a maximal density, a lower/higher ratio results in a more loosely crosslinked network (Mũnoz et al., 2014; Greene and Lin, 2015; Van Hoorick et al., 2018). Parmentier et al. evaluated the influence of the type and distribution of crosslinkable moieties on the biophysical cues targeting osteogenesis by comparing extrusion bioprinted GelMA (5 w/v%, DS 99%), GelNB/GelSH (5 w/v%, DS 91%/67%, thiol:ene = 1:1) and GelNBNB/GelSH (5 w/v%, DS 169%/67%, thiol:ene = 1:1) encapsulating 1 million human DPSCs per mL (Parmentier et al., 2023). An excellent CAD-CAM mimicry was obtained for all inks. However, the biocompatible irradiation dose (4.8 mJ/cm^2^) resulted in a large discrepancy in reacted functionalities between the thiol-ene inks (almost 100% conversion) and GelMA (about 50% conversion). Consequently, the mesh size of the GelMA crosslinked network is expected to be larger as compared to the thiol-ene inks, proven by its larger mass swelling ratio and lower compressive modulus. The higher DS of GelNBNB and similar conversion compared to GelNB, indicate a lower mesh size for crosslinked GelNBNB/GelSH which was confirmed by the lower mass swelling ratio and higher compressive modulus compared to crosslinked GelNB/GelSH. The higher network density for crosslinked GelNBNB/GelSH and GelNB/GelSH compared to crosslinked GelMA, shifted the compressive modulus into the range for optimal osteogenesis of encapsulated stem cells (11–30 kPa) (Huebsch et al., 2010). The increased compressive modulus facilitates RGD-ligand clustering and thereby activates osteogenic differentiation pathways (Huebsch et al., 2010; Chaudhuri et al., 2016). Moreover, the crosslinked thiol-ene systems have a higher viscoelasticity enabling mechanical cell-mediated matrix remodeling facilitating RGD-ligand clustering (Chaudhuri et al., 2016). In earlier work, the reduced viscoelasticity of crosslinked casted GelMA was explained by the kinetic chains that highly restrict the network mobility (Parmentier et al., 2024). Those results for non-printed hydrogels encapsulating cells are consistent with the reported inks, for which increased ALP activity (day 7 and 14), calcium deposition (day 21) and cell spreading (i.e. indicated by an increasing aspect ratio and decreasing circularity on day 1) were found at increased viscoelasticity along with a compressive modulus within the range for optimal osteogenesis (Parmentier et al., 2024). Finally, a similar cell viability was obtained compared to extrusion bioprinted GelNB/GelSH inks encapsulating ASCs (Burchak et al., 2022). However, this research did not assess the osteogenic differentiation post-printing (Burchak et al., 2022). Overall, those experiments reveal the potential of gelatin-based thiol-ene bioinks serving osteogenesis.

##### 2.1.2.2 Schiff base

A second step-growth system exploits the Schiff base formation between amino and aldehyde groups. Upon blending (macro-) molecules with both functionalities, spontaneous crosslinking occurs resulting in reversible bonds, which dissociate and re-associate when external or cellular forces are applied (Wang and Heilshorn, 2015; Yang et al., 2021).

The ink consisting of oxidized alginate (ADA) and gelatin (Gel), known as ADA-Gel, is one of the scarce bioinks reported for bone tissue engineering exploiting step-growth crosslinking. ADA is obtained by partially oxidizing alginate’s hydroxyl groups into aldehydes using sodium periodate. During oxidation, alginate partially degrades resulting in an enhanced biodegradability (Liang et al., 2011; Reakasame and Boccaccini, 2018). By increasing the oxidation degree or increasing the ADA:Gel ratio, the crosslinking degree increases, resulting in a denser network (Sarker et al., 2014; Zehnder et al., 2015; You et al., 2020). Additionally, the cell-interactivity of alginate inks is improved by combining ADA with gelatin, allowing cell adhesion (Grigore et al., 2014; Sarker et al., 2014; Zehnder et al., 2015). Under culture conditions, (uncrosslinked) gelatin is partially released facilitating cell migration, proliferation and differentiation (Balakrishnan and Jayakrishnan, 2005; Sarker et al., 2014; Zehnder et al., 2015). In general, by increasing the ADA:Gel ratio, the release of gelatin is reduced (Boanini et al., 2010). However, the spontaneous imine bond formation makes the mechanical properties time-dependent, limiting the processing of the ink to a specific time-window. As illustrated by Zehnder et al. and Leite et al., the processing window varies depending on the specific composition of the ink (i.e. between 5 and 60 min of crosslinking time) (Zehnder et al., 2015; Leite et al., 2016).

Two different crosslinking strategies were applied in combination with the spontaneous imine bond formation encompassing solely physical gelation or a combination of physical and chemical gelation. The first strategy is performed using 0.1 M CaCl_2_ for 10 min and results in ionic interactions between Ca^2+^, a divalent cation, and negatively charged carboxylic acids. Zehnder et al. targeted an osteoid-mimicking construct by determining an optimal ADA-Gel concentration and cell suspension (Zehnder et al., 2017). After bioplotting, the construct was physically crosslinked using CaCl_2_ (10 min, 0.1 M). 7.5 w/v% ADA-Gel (oxidation degree not specified) was selected following an evaluation comparing the nanoscale stiffness with the stiffness identified for optimal osteogenesis after cell seeding (2D, 25–40 kPa) (Engler et al., 2006). Since the goal was to encapsulate cells (3D), it would have been more suitable to quantify the compressive modulus and to compare it with the range identified for optimal osteogenesis after cell encapsulation (3D, 11–30 kPa) (Huebsch et al., 2010). A co-culture of murine osteoclast (RAW.264) and murine osteoblast (ST-2) progenitor cells were used to recapitulate the dynamic crosstalk between osteoclasts and osteoblasts during bone formation and resorption (Detsch and Boccaccini, 2015). The cell density was 2 million cells/mL with ST2:RAW equal to 100:1. The co-culture was selected due to the higher OPN concentration (day 21), which indicates osteoblastic differentiation, higher tartrate resistant acid phosphatase (TRAP) activity (day 21), which indicates osteoclast differentiation, and higher vascular endothelial growth factor (VEGF) release (day 21), which promotes angiogenesis, with respect to corresponding monocultures without the use of differentiation factors.

**FIGURE 3:**
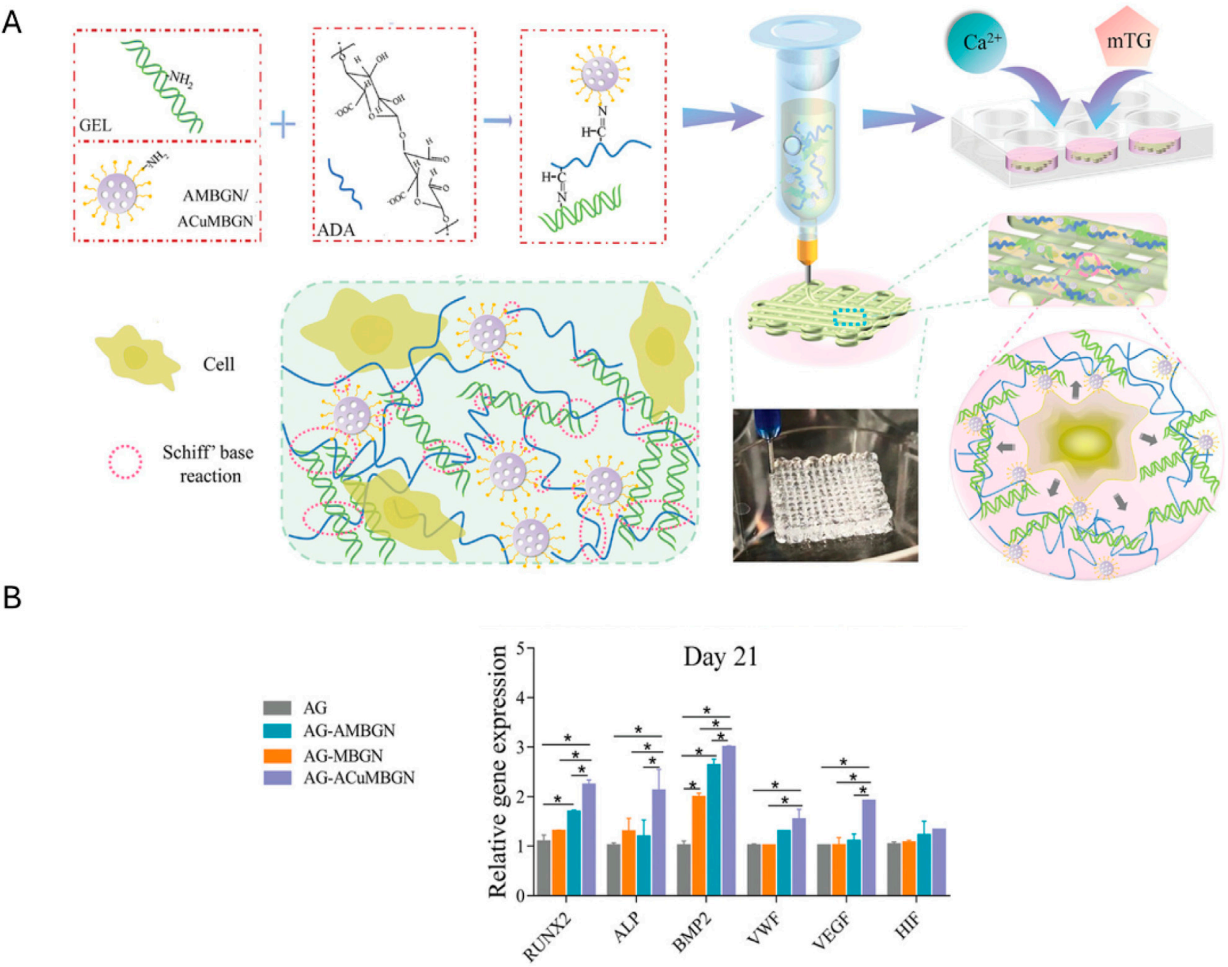
**(A)** Graphical representation of the extrusion-based bioprinting process of the Schiff base bioink consisting of Gel (gelatin), ADA (oxidized alginate), ACuMBGN (aminated copper-doped mesoporous bioactive glass nanoparticles), mouse BMSCs (bone marrow-derived stem cells, 1 million cells/mL) and the different crosslinkers (including CaCl_2_ and microbial transglutaminase (mTG)) for post-crosslinking. **(B)** The relative expression of osteogenesis-related (RUNX2, ALP and BMP2) and angiogenesis-related (VWF, VEGF and HIF) genes by BMSCs in bioprinted ADA-Gel (AG), AG containing mesoporous bioactive glass nanoparticles (AG-MBGN), AG containing aminated MBGN (AG-AMBGN) or AG containing ACuMBGN (AG-ACuMBGN) on day 21. Reproduced from Zhu et al. (2022) under open access license.

Besides performing just a physical gelation step after bioplotting, a chemical gelation step using microbial transglutaminase (2.5–10 w/v%, 10–15 min), inducing the step-growth bond formation between the epsilon amino-group in lysine and the gamma-carbonyl on glutamine, has also been performed (Chen et al., 2005). Zhu et al. bioplotted ADA-Gel (Gel 12 w/v% and ADA 3 w/v% were mixed, oxidation degree 30%) with different types of mesoporous bioactive glass nanoparticles (MBGNs) including copper-doped MBGNs (CuMBGNs) and aminated copper-doped MBGNs (ACuMBGNs) to generate a micro-environment stimulating osteogenic and angiogenic differentiation and to improve cell adhesion and spreading (Figure 3) (Zhu et al., 2022). The delivery of biologically active ions including calcium and silicon stimulated osteogenic differentiation proven by the RUNX2, ALP and BMP-2 expression (day 21). Additionally, it was hypothesized that the delivery of Cu^2+^ cations induced angiogenesis indicated by VEGFA and von Willebrand factor (VWF) expression (day 21). Although the immunofluorescence staining and RT-qPCR showed an enhanced osteogenic gene expression when encapsulating mouse BMSC (1 million cells/mL) in ADA-Gel with ACuMBGNs, the compression modulus was about 100–150 kPa, which is rather stiff to allow RGD ligand clustering by matrix deformation (*vide supra*) (Huebsch et al., 2010). Zhu et al. explained the effect on osteogenesis due to the dynamic nature of the network. Indeed, the presence of reversible covalent imine bonds and ionic interactions results in a visco-elastic matrix, mechanically re-modellable through cellular forces allowing the cleavage and formation of existing and new reversible bonds respectively (Chaudhuri et al., 2016; Yang et al., 2021). In contrast, Chaudhuri et al. reported high osteogenic differentiation when using an ionically crosslinked (non-printed), viscoelastic alginate matrix with an elastic modulus of 17 kPa (Chaudhuri et al., 2016). Thus, more research is required to prove that the dynamic nature of the network is responsible for the observed osteogenic differentiation rather than the MBGNs and/or the degradability of gelatin.

**FIGURE 4:**
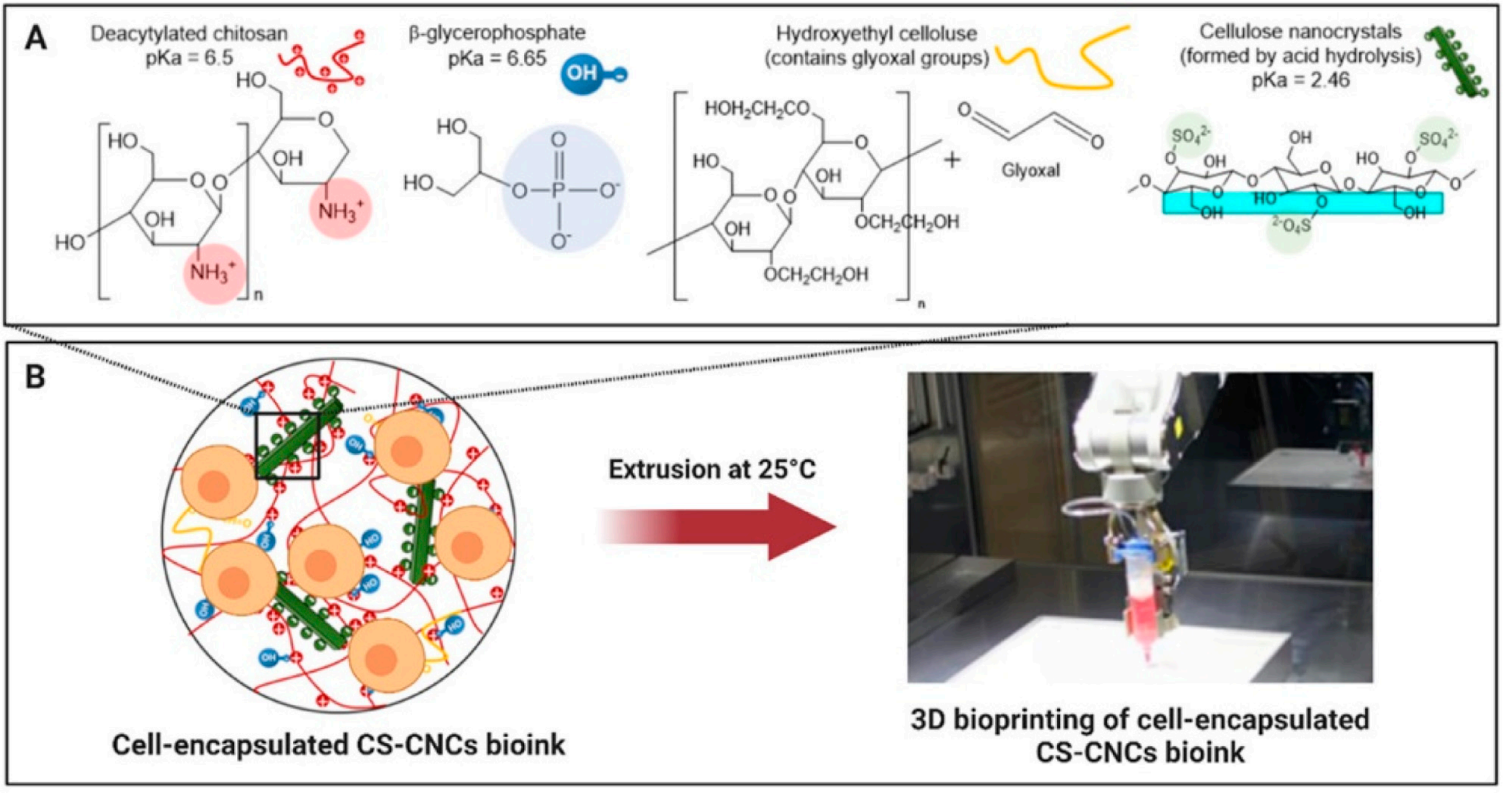
The Schiff base bioink containing chitosan (CS), β-glycerophosphate, hydroxyethyl cellulose, cellulose nanocrystals (CNC), glyoxal **(A)** and pre-osteoblastic murine MC3T3-E1 (5 million cells/mL) is subsequently bioprinted through extrusion at 25°C **(B)**. Reproduced from Maturavongsadit et al. (2021) under open access license.

Another bioink exploiting Schiff base formation contained chitosan (85% deacetylated, 2 w/v%), β-glycerophosphate (BGP, 0.1 M), hydroxyethyl cellulose (HEC, 0.1 mg/mL, glyoxal not quantified), cellulose nanocrystals (CNC, 1.5 w/v%) and MC3T3-E1 (5 million cells/mL) as illustrated in Figure 4 (Maturavongsadit et al., 2021). BGP and HEC were added to promote gel formation at 37°C, neutral pH and to enhance shape retention respectively (Chenite, 2001). The former is caused by purely physical interactions between chitosan chains (Chenite, 2001; Wang and Stegemann, 2011). The latter is obtained through the Schiff base formation between chitosan’s amines and the dialdehyde crosslinker glyoxal present in HEC (Hoemann et al., 2007; Wang and Stegemann, 2011). CNC improved the storage modulus, Young’s modulus and viscosity mainly through hydrogen bonding with chitosan chains (Maturavongsadit et al., 2020). After extrusion bioprinting at 25°C, the scaffolds were incubated at 37°C causing fast gelation (< 7 s). The osteogenic gene expression revealed a faster onset of osteogenesis (i.e. peak on day 7) when using 1.5 w/v% CNC compared to the lower concentrations based on the ALP activity. Moreover, this system exhibited the highest ECM formation, mineralization (on days 7, 14 and 21) and calcium deposition (on days 14 and 21). The osteogenic differentiation improved with increasing storage and Young’s moduli, which can be explained by the improved RGD-ligand clustering (*vide supra*).

##### 2.1.2.3 Enzymatic crosslinking

**FIGURE 5:**
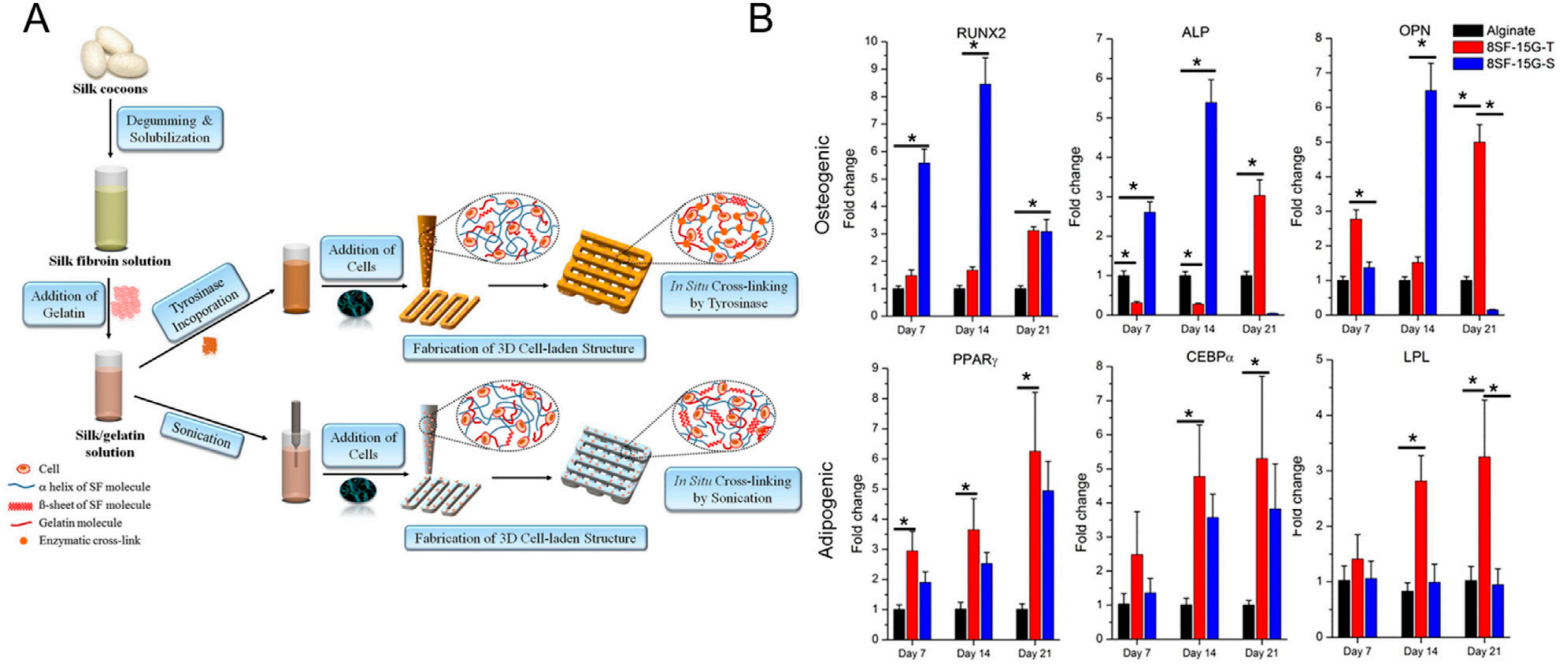
**(A)** Schematic representation of extrusion bioprinting of silk fibroin-gelatin (SF-G) constructs starting from silk cocoons. The bioink containing 8 w/v% SF, 15 wt% gelatin and 2 – 5 million human nasal inferior turbinate tissue-derived mesenchymal stromal cells (hTMSCs) was physically or chemically crosslinked via sonication (8SF-15G-S) or mushroom tyrosinase (8SF-15G-T). **(B)** Evaluation of the relative expression of osteogenesis-related (RUNX2, ALP and OPN) or adipogenesis-related (PPAR_γ_, CEBP_α_ or LPL) genes by bioprinted hTMSCs. Reproduced from Das et al. (2015) with permission.

Besides using enzymes (e.g. microbial transglutaminase) in combination with other crosslinking systems (e.g. ADA-Gel: Schiff base), enzymes as such, including transglutaminase and mushroom tyrosinase, were also utilized to induce covalent crosslinking of bioinks targeting osteogenesis (Das et al., 2015; Chawla et al., 2018; Sharma et al., 2019; Zhu et al., 2022; Kara Özenler et al., 2023; 2024; Bider et al., 2024). Pragnere et al. developed an *in vitro* system to differentiate primary osteoblasts (0.3 million cells/mL) into osteocytes within a hydrogel constituting gelatin (5 w/v%), fibrinogen (2 w/v%) and alginate (1 w/v%) (Pragnere et al., 2025). A varying multivalent crosslinking strategy was exploited with transglutaminase (0.2 vs. 4 w/v%) and calcium ions (0.02 vs. 3 w/v%) to obtain hydrogels with a similar stiffness but different viscoelastic behavior as well as hydrogels with similar viscoelastic behavior but different stiffnesses. The most viscoelastic ink (i.e. tan (delta) = 0.13) with the lowest compressive modulus (8.6 kPa) resulted in the highest hydrogel contraction, characteristic cell proliferation evolution and stabilization, highest ECM production, transition of cuboidal to dendritic morphology, and fastest expression of the osteocyte specific marker phosphate regulating natural endopeptidase on the X chromosome (PHEX), indicative of osteoblasts transitioning into osteocytes. The ink with a similar viscoelastic behavior (tan (delta) = 0.09) yet higher compressive modulus (15.5 kPa) impeded the differentiation towards osteocytes through reduced degradability. Another study used mushroom tyrosinase to covalently crosslink gelatin and silk, via phenol coupling, Michael-Type addition or Maillard reaction, causing long-term stability under culture conditions (Chen et al., 2002; Freddi et al., 2006; Das et al., 2015). A blend of silk fibroin (SF, 8 w/v%) and gelatin (Gel, 15 wt%) encapsulating BMSCs (2-5 million cells/mL) was physically crosslinked via sonication (10 s at 50% amplitude) or chemically crosslinked using tyrosinase (500 U) prior to extrusion bioprinting as illustrated in Figure 5A (Das et al., 2015). The former induces β-sheet formation in SF resulting in a more tightly packed matrix (Wang et al., 2008). The lower number of β-sheets in the chemically crosslinked hydrogel possibly results in a less compact matrix easier re-modellable by cells, as confirmed by the higher swelling, lower stiffness and higher proliferation. On days 7 and 14, the collagen production as well as osteogenic gene expression (i.e. RUNX2, ALP and OPN) was higher in case of sonication, potentially caused by an increased stiffness due to a higher fraction of β-sheets (Figure 5B). Leaching of uncrosslinked gelatin potentially caused the lower gene expression on day 21. It is worth mentioning that SF bioinks as such also improve osteogenic differentiation and mineralization by upregulating the β-catenin expression and suppressing the Notch signaling pathway (Chawla et al., 2018). Additionally, the amorphous connections between β-sheets provide nucleation sites for hydroxyapatite deposition (Marelli et al., 2012; Vetsch et al., 2015). Besides, also adipogenic gene expression was evaluated in both SF-Gel deposited bioinks. In correspondence with previous reports, the tyrosinase crosslinked SF-Gel bioink, which is less favorable towards osteogenic differentiation, had upregulated adipogenic gene expression (i.e. peroxisome proliferator-activated receptor gamma (PPAR-γ) and lipoprotein lipase (LPL)) on days 14 and 21 as illustrated in Figure 5B. Thus, the sonicated SF-Gel bioinks support more osteogenic differentiation, however the uncrosslinked gelatin prevents long-term stability under culture conditions. Other studies reporting on extrusion-based bioprinting of tyrosinase crosslinked SF-Gel bioink encompassed a two-step culturing approach simulating endochondral ossification or used the sustained release of calcium to improve osteogenic differentiation (Chawla et al., 2018; Sharma et al., 2019). The latter approach resulted in a higher osteogenic gene expression potentially promoted by the improved stiffness caused by the higher β-sheet fraction (Dubey et al., 2015).

##### 2.1.2.4 Small molecules as crosslinker

A final type of crosslinking involves the use of genipin, a natural crosslinker obtained from gardenia fruit. A bioink composed of collagen (5 wt%) and ASCs (1 million cells/mL) was crosslinked in a genipin bath (1 mM) for 1 h after extrusion bioprinting (Kim et al., 2016). The potential of the collagen scaffolds towards osteogenesis was compared to a similar CaCl_2_ crosslinked alginate (5 w/v%) bioink encapsulating ASCs (1 million cells/mL). A higher metabolic activity, cell density, (days 14 and 21), ALP activity (days 7 and 14) and calcium deposition (days 7 and 14) were reported in case of the collagen-based bioink. Although the reported ALP activity and calcium deposition were significantly higher compared to those of the alginate bioink, the results should be normalized for the different cell densities on days 14 and 21 to allow a fair comparison. However, the RT-qPCR proves the significantly increased expression of BMP-2, RUNX2, Col1 and OCN on day 28 in the collagen bioink. Since different genes are maximally expressed at different stages of the differentiation process, a time course of the gene expression would provide more insights. A potential explanation for the improved osteogenic differentiation is related to the absence of cell-adhesive motifs and MMP-degradable motifs in alginate, since both strongly correlate to osteogenic differentiation (*vide supra*) (Huebsch et al., 2010; Khetan et al., 2013; Chaudhuri et al., 2016).

### 2.2 Inkjet bioprinting of constructs targeting osteogenesis

Inkjet bioprinting, a deposition-based biofabrication technique, entails the precise deposition of cell-laden droplets according to a computer-aided-design (CAD), thereby resulting in 3D cellular constructs (Lorber et al., 2013; Xu et al., 2013; 2019). Continuous inkjet printing and DoD are the main types of inkjet printing (Li et al., 2020). In the former type, a piezoelectric crystal causes the nozzle to vibrate, ensuring a continuous stream of ink through the nozzle (Li et al., 2015). Droplets are continuously formed according to the Rayleigh-Plateau instability, even though the droplets are not contributing to the print (Li et al., 2020). A potential difference between the nozzle and the substrate charges the droplets, enabling their deflection when passing through charged deflectors (Derby, 2010). In this way, the unneeded droplets are separated from the desired ones (Derby, 2010). Subsequently, this captured ink is sent back to the printhead to be re-used (Derby, 2010; Saunders and Derby, 2014; Alamán et al., 2016; Kumar et al., 2021). The final droplet position is regulated by controlling the movement of the droplets and the position of the substrate (Derby, 2010). The use of continuous inkjet bioprinting is limited due to the printer’s complexity (i.e. droplet charging, deflection and recycling system) and the contamination risk if droplets are re-used (Saunders and Derby, 2014; Li et al., 2015; 2020). DoD is another type of non-contact deposition-based printing exclusively producing droplets when the actuator is activated (Romagnoli et al., 2016; Li et al., 2020). The actuator induces a thermally or mechanically generated pressure pulse resulting in picolitre droplets with a 15–100 µm diameter and a long tail rupturing into the primary droplet followed by satellite droplets (Wallace and Grove, 2003; Derby, 2010; Romagnoli et al., 2016; Li et al., 2020). If the droplets are not merged prior to the impact on the substrate, a non-circular impact is caused lowering the resolution and accuracy (Derby, 2010). This phenomenon, called droplet splashing, is also caused by the high-speed droplet impact and should be controlled when printing µm-scale constructs (Li et al., 2020). When the actuator is not activated, the fluid remains within the fluid chamber due to surface tension (Derby, 2010). Thermal and piezoelectric DoD are the most prevalent inkjet techniques (Li et al., 2015).

DoD exhibits potential for biofabrication applications due to its high throughput, non-contact and drop-on-demand printing. The maximal throughput depends on the number of nozzles (up to hundreds) and the ejection frequency (up to 250 kHz) and can be up to 80 mL/h/printhead (Xu et al., 2006; Wijshoff, 2010; Cui et al., 2012a; Li et al., 2020). By using multiple nozzles, diverse bioinks with different cell types can be printed within a single construct. Non-contact printing lowers the contamination risk hence allows *in situ* printing (Cui et al., 2012b; Li et al., 2020). Moreover, it prevents the deformation of previously deposited structures (Li et al., 2020). Lastly, the computer-controlled drop-on-demand printing allows precise spatial and temporal control. However, its use is limited due to the low viscosity requirement (<10 mPa.s) to avoid clogging and the low cell concentration (∼1 million cells/mL) (Murphy and Atala, 2014; Hölzl et al., 2016; Li et al., 2020). An important limitation due to the low ink viscosity is cell sedimentation resulting in an increase in cell density at the bottom of the printhead and subsequently cell aggregation (Liu et al., 1970; Xu H. et al., 2022). This phenomenon results in a non-uniform cell distribution, unstable droplet formation and nozzle clogging (Lorber et al., 2013; Xu H. et al., 2022). Different solutions have been applied associated with pros and cons including active bioink stirring, bioink manipulation to obtain neutral buoyance and active bioink circulation (Liu et al., 2022; 2023; Xu H. et al., 2022; Liu and Xu, 2024). Additionally, cells adhere to the inner surfaces of the printing set-up by Van der Waals forces, resulting in constriction and clogging as well as a lower cell number with respect to the theoretical number (Dersoir et al., 2015; Sendekie and Bacchin, 2016; Ng and Shkolnikov, 2024).

#### 2.2.1 Thermal drop-on-demand inkjet bioprinting

In thermal DoD, the thermal actuator heats the ink for a short duration (∼µs) resulting in the formation of heat bubbles at the resistor (Cui et al., 2010; Murphy and Atala, 2014; Li et al., 2015; 2020). The expansion of the bubbles drives the ejection of the ink and the formation of droplets (Li et al., 2020). After heating, the ink cools down by heat transfer causing the heat bubbles to collapse and hence, a pressure is induced to refill the printhead (Li et al., 2015). The diameter of the produced droplets (30–80 μm, 150–200 pL) is similar to the nozzle diameter (50 µm) (Xu et al., 2006; Cui et al., 2010; Tirella et al., 2011; Li et al., 2015; 2020). The ink should be vaporizable and thermally stable (Li et al., 2015; Gilani et al., 2023).

Generally, the influence on cell viability and functionality is limited (Xu et al., 2005; Xu et al., 2006; Cui et al., 2010; Xu et al., 2013). A reduction in cell viability can be caused by thermal and mechanical stress (Kumar et al., 2021). During printing, the ink’s temperature increases with 4°C–10°C, while the thermal actuator reaches temperatures up to 300°C (Cui et al., 2010; 2012b). Hence, only cells in close vicinity to the heater experience a critical heat shock (Kumar et al., 2021). Additionally, mechanical stresses exerted during the printing process and upon droplet impact cause cell damage and cell death (Ng et al., 2021). Simulations performed by Sohrabi et al. showed that mechanical deformation, when forcing cells through the nozzle, rather than the temperature increase, results in transient pores, which are repaired within hours (Sohrabi and Liu, 2018). Ng et al. observed an increasing cell viability when decreasing the impact velocity along with the preservation of normal cell morphology, high cell viability and cell proliferation post-printing when controlling the droplet velocity and volume (Ng et al., 2021). Finally, they observed a reduced cell number within the cell-suspension droplet compared to the theoretical number, attributed to adherence to the inner wall (Ng et al., 2021). Xu et al. evaluated the viability, proliferation rate and phenotype of smooth muscle cells, endothelial cells and human osteogenic stem cells respectively in both printed (i.e. cells dispensed in a CaCl_2_ solution were introduced into alginate and collagen solutions using thermal DoD) and non-printed (i.e. seeded) samples (Xu et al., 2013). No differences in viability, proliferation nor in osteogenic phenotype were noticed (Xu et al., 2013). However, just a single antibody (i.e. anti-octamer-binding transcription factor 4 (anti-OCT 4)) was used for the phenotype evaluation (Xu et al., 2013). Solis et al. performed a more detailed analysis and reported the altered gene expression due to thermal DoD of cells dispensed in a CaCl_2_ solution. They quantified the overexpression of cytokines including VEGF-A and heat shock proteins (HSPs), that may influence angiogenesis, in printed endothelial cells with respect to manually pipetted cells (Solis et al., 2019). Morales et al. reported the strain-induced temporary auto-initiated reprogramming (SITAR) of printed fibroblasts suspended in phosphate buffered saline (PBS) resulting in the temporary up-/downregulation of genes corresponding to pluripotent stem cells (Ablanedo Morales et al., 2023). Additionally, they cultured thermally DoD printed fibroblasts using a cardiomyocyte differentiation protocol resulting in cardiomyocyte-like morphology and troponin I type 3 expression (Ablanedo Morales et al., 2023). They hypothesized that the stretching of cells through the nozzle evoked this temporary pluripotent response since manually dispensed cells through the same orifice (without heat) also resulted in the expression of the pluripotent markers (Ablanedo Morales et al., 2023). Therefore, although the cellular viability is preserved, the printing process influences the gene expression, evoking the need for more dedicated research at the genome level (Xu et al., 2005; Xu et al., 2006; Cui et al., 2010; Xu et al., 2013; Solis et al., 2019; Ablanedo Morales et al., 2023).

Gao et al. employed a modified HP Deskjet 500 as thermal inkjet printer to deposit a bioink containing exclusively poly (ethylene glycol)-dimethacrylate (PEGDMA, degree of methacrylation not specified) or PEGDMA together with GelMA (DS not specified), or PEGDMA together with acrylated GRGDS-peptide (1 mM) and acrylated MMP-sensitive peptides (1 mM) (Gao et al., 2015b; Gao et al., 2015a). Human BMSCs were encapsulated in the inks at a final density of 6 million cells/mL (Gao et al., 2015b; Gao et al., 2015a). PEGDMA was selected based on its macroscopic mechanical properties mimicking more appropriately the mechanical properties of bone with respect to natural hydrogels. However, due to the absence of cell-adhesion peptides and MMP-sensitive degradation sites as well as its low protein adsorption, this inert biomaterial does not facilitate cell adhesion, degradation, migration and spreading (Horbett, 1994; Burdick and Anseth, 2002; Nichol et al., 2010). Nevertheless, the synthetic nature of PEG allows to tune the biological behavior through the controlled introduction of bioactive peptides/proteins (Yang et al., 2005; Gill et al., 2012). The introduction of MMP-sensitive and cell-adhesion peptides enables cell-mediated matrix degradation, shifting the elastic matrix towards a more viscoelastic matrix allowing RGD-ligand clustering (Yang et al., 2005; Gill et al., 2012; Schultz et al., 2015; Chaudhuri et al., 2016). Consequently, signaling pathways are activated associated with osteogenic differentiation (Chaudhuri et al., 2016). Hence, GelMA, containing both corresponding peptide sequences, or the incorporation of MMP-sensitive peptides and the RGD sequence are essential in the PEGDMA ink to target osteogenic differentiation (Nichol et al., 2010). This is proven by the results of Gao et al. indicating an increased expression of osteogenic genes (e.g. RUNX2, ALP and OCN) in both RGD-containing, MMP-cleavable inks as compared to conventional PEGDMA (Gao et al., 2015b; Gao et al., 2015a). Moreover, the compressive modulus increased significantly after 21 days of culturing in osteogenic differentiation medium proving osteogenic differentiation and ECM production (i.e. collagen) (Gao et al., 2015b; Gao et al., 2015a). Despite the important influence of MMP-sensitive peptides and RGD on the cellular behavior, no degradation study nor cell morphology assessment were performed. A sound comparison of both RGD- and MMP-sensitive peptide-containing inks is not feasible due to the absence of data regarding the quantity of MMP-sensitive peptides and RGD moieties incorporated in the inks.

#### 2.2.2 Piezoelectric drop-on-demand inkjet bioprinting

In piezoelectric DoD, the piezoelectric actuator suddenly deforms the fluid chamber when receiving an electrical signal resulting in a pressure/acoustic wave through the ink (Tekin et al., 2008; Li et al., 2020). When the kinetic energy is larger than the surface energy needed to create a droplet, a droplet is ejected (Derby, 2010). The acoustic frequencies evoked during this process are generally situated between 1–20 kHz and correspond to the frequencies leading to cell wall damage and lysis (Seetharam and Sharma, 1991; Derby, 2010; Li et al., 2020). In the absence of the electrical signal, the fluid chamber’s original shape is restored/maintained (Li et al., 2015). Both a hybrid and a single actuation mode, including squeeze, bend, shear, push and needle collision, exist (Li H. et al., 2019). The diameter of the produced droplets (50–100 μm, 150 pL) is similar to the nozzle diameter (18–120 µm) (Wijshoff, 2010; Christensen et al., 2015; Li et al., 2015; 2020). Due to the absence of extensive heating, more inks are compatible as well as a longer printhead lifetime is obtained (Li et al., 2015).

Generally, the influence on cell viability and functionality is limited (Saunders et al., 2008; Mau et al., 2015; Negro et al., 2018; Kumar et al., 2021). A reduction in cell viability as compared to unprinted cells is evoked by the mechanical stress exerted during the printing process or upon droplet impact (Shi et al., 2018). Shear stress inducing cell deformation and cell death can be minimized by controlling the ink’s viscosity, the nozzle shape and the voltage waveform amplitude (Ning et al., 2020; Xu H.-Q. et al., 2022). Lorber et al. investigated the impact of piezoelectric DoD on cell shape, number and phenotype by comparing unprinted and printed cells dispensed in culture medium (Lorber et al., 2013). They concluded that, despite the high shear rate and acceleration, no significant deformation, active cell disintegration nor phenotype change occurred (Lorber et al., 2013). However, a reduction in cell number was noticed after printing due to cells adhering to the internal parts of the printer set-up (e.g. printhead and nozzle) (Saunders et al., 2008; Parsa et al., 2010; Chahal et al., 2012; Yamaguchi et al., 2012; Ferris et al., 2013; Lorber et al., 2013). Barui et al. investigated the influence of the actuating voltage on the proliferation rate and membrane integrity of cells dispensed in phosphate buffered saline (Barui et al., 2020). They reported a reduced proliferation rate as compared to unprinted cells. Moreover, although a higher voltage allows easier stable droplet formation, a lower membrane integrity (i.e. higher membrane porosity) was obtained (Barui et al., 2020). Yumoto et al. performed a transcriptome analysis revealing a non-significantly different gene expression between manually dispensed and piezoelectrical inkjet-printed mouse embryonic stem cells dispensed in medium (Negro et al., 2018; Yumoto et al., 2020; Kumar et al., 2021). However, also here, a more in-depth transcriptome analysis is needed to determine the impact of piezoelectric inkjet bioprinting on the phenotype.

Burchak et al. used a piezoelectric printer to deposit three different GelNB/GelSH formulations encapsulating human ASCs (2 million cells/mL) (Burchak et al., 2022). The formulations exhibited significantly different storage and compressive moduli by using different degrees of substitution, thiol:ene ratio and final gelatin concentration. Prior to printing, the differentiation capabilities of the bioinks towards the osteogenic lineage were evaluated. Similar to Huebsch et al., the stem cells exhibited maximal commitment towards osteogenesis when the commitment towards the adipogenic lineage was minimal and a correlation between the compressive moduli and osteogenic differentiation was identified (Huebsch et al., 2010). However, although an intermediate compressive modulus is expected to result in maximal osteogenic differentiation (*vide supra*), here the maximal storage modulus resulted in the most promising cell response. Higher compressive moduli of a similar ink, e.g. by increasing the degree of substitution (DS), could be evaluated to assess if a similar (biphasic) relation between the compressive modulus and osteogenic differentiation exists as reported earlier. Afterwards, the ink with the highest commitment towards osteogenesis was piezoelectrically printed resulting in an acceptable cell viability (around 80% post-printing). Although this paper proves the ability to print GelNB/GelSH with a piezoelectric inkjet printer to serve bone tissue engineering, the commitment towards osteogenesis was not assessed post-printing. Moreover, it should be noted that high photo-initiator concentrations (3–10 mM LAP) were used without performing any crosslinking assessment. The photo-initiator concentration should be minimized to prevent damage to DNA and proteins induced by free radicals (Rehmann and Kloxin, 2013). Previous literature set the cytotoxic limit of LAP to 1.12 mM for cell encapsulation applications (Markovic et al., 2015). Moreover, similar GelNB/GelSH inks were printed/casted using about 80 times lower amounts of LAP with respect to the crosslinkable ene-moieties (Van Damme et al., 2021; Van Hoorick et al., 2021; Parmentier et al., 2023; Parmentier et al., 2024). Hence, the ideal concentration should be identified by evaluating the crosslinking efficiency and crosslinking kinetics using photo-rheology, gel-fraction experiments and high-resolution magic angle spinning proton nuclear magnetic resonance (HR-MAS ^1^H-NMR) spectroscopy.

## 3 Light-based bioprinting of constructs targeting osteogenesis

Mainly two modified natural polymer resins have been used to mimic the non-mineralized collagenous bone osteoid through light-based bioprinting, including silk fibroin and gelatin (Table 3). Both fibrous proteins have been favorably used since the nucleation of minerals is stimulated within their structure hereby mimicking the mineralizing ability that collagen type I has within the native bone ECM (Kuttappan et al., 2016; Midha et al., 2016).

Cell encapsulation within these resins necessitates not only cytocompatibility of all resin components but also mild reaction conditions such as a favorable light wavelength and dose that prove to be unharmful towards the viability and functionality of cells (Levato et al., 2023). The light-based bioprinting field employs a wide range of light wavelengths to create constructs facilitating osteogenesis going from the ultraviolet A range (UV-A, 315–400 nm), towards the visible light range (VIS, 380–760 nm) and up to the near infrared (NIR, 760 nm–1,400 nm) with the wavelength ranges specified according to the ISO 21348 standard. Shorter wavelengths in the UV-A range inherently carry a higher energy which might impede encapsulated cell viability and function through the generation of free radicals inducing indirect DNA damage (Wong et al., 2015). Nevertheless, when a low dose (5.25 J/cm^2^) was used, gene expression was mainly influenced by the micro-environment (2D versus 3D culture, chain-versus step-growth 3D encapsulation and associated number of radicals present) rather than the UV-A exposure itself (Wong et al., 2015).

In contrast to the UV-A range, starting from, but not limited to, a dose in the same order of magnitude, effects on cell viability, proliferation and differentiation are observed when increasing the wavelength to visible or near infrared light. In this regard, the blue and green light range have been found to upregulate the osteogenic differentiation and downregulate the proliferation of human ASCs through an enhancement of the intracellular calcium content and reactive oxygen species levels while reducing the cellular adenosine triphosphate concentration and lowering the intracellular pH in contrast with the red and near infrared region where the opposite trend was observed (Wang et al., 2016; 2017). The same trend was seen for human periodontal ligament stem cells where only the red and near infrared region were studied. A higher differentiation was observed in the lower wavelength red region whereas the cells showed a higher proliferation capacity when the wavelength was increased (Chaweewannakorn et al., 2021). In contrast, another study evaluated only near infrared irradiation of human BMSCs and reported not only an increased proliferation but also an enhanced dose-dependent neuro- and osteogenic differentiation with higher doses causing a higher extent of stimulation (Soleimani et al., 2012). Given the inversely proportional energy-wavelength relationship outlined before, a wider range of biocompatible doses might be achievable when using higher wavelength light which allows to more easily tune the irradiation dose for maximum differentiation and maximum proliferation while keeping the wavelength constant (Emelyanov and Kiryanova, 2015). In this context, blue and near infrared light have been successfully combined in enhancing osteogenic differentiation of human DPSCs through activated mitochondrial biogenesis (Kim et al., 2023). In general, it should be taken into account that, when using light, the reported cellular outcomes are highly dependent on the dose (hereby corrected for possible beam divergence from the used light irradiation set-up), the frequency of exposure and the investigated cell type since, even for visible and near infrared light, large doses can inhibit or even kill cells, hence explaining why the lowest (0.05 J/cm^2^, subthreshold stimuli phase) and highest (42 J/cm^2^, photoshock) doses for human dental pulp stem cells showed no measurable effects (Emelyanov and Kiryanova, 2015; Kulkarni et al., 2020).

Based on the different light-based bioprinting techniques currently used within the biofabrication field serving osteogenesis, an overview is first given of each bioprinting technique whereafter the different biophysical cues targeting osteogenesis within these constructs and their effect on the osteogenic differentiation of the encapsulated cells, are discussed.

### 3.1 Stereolithography- and digital light processing-based bioprinting

Both printing methods, stereolithography (SLA) and digital light processing (DLP), project UV- or visible light patterns of the discretized and sliced CAD in a point-by-point and layer-by-layer fashion respectively onto the photo-crosslinkable resin. After one layer is finished, the motorized build platform moves away vertically to allow the uncured resin to flow back whereafter the print head is repositioned to allow crosslinking of the subsequent layer. These printing techniques mainly differ in the way the light is patterned with SLA using raster laser scanning whereas DLP uses either a digital mirror device (DMD) or a liquid crystal display (LCD) projection system (Li W. et al., 2023). Overall, the outcome of the bioprinting processes DLP and SLA in terms of printability, printing time, attainable sample size, resolution, shape fidelity and print stability is mainly affected by the constituents of the bioresin (i.e. photo-crosslinkable polymer concentration and reactivity, cell type and concentration, concentration and efficiency of photo-initiators, -absorbers and/or -inhibitors), the light projection method, sample post-processing and the delivered light dose to the resin through variations in the light intensity and exposure time (Liang et al., 2021; Goodarzi Hosseinabadi et al., 2022; Levato et al., 2023; Li W. et al., 2023).

The LCD/DMD of the DLP can cure an entire layer at once, making the DLP process faster than the point-by-point crosslinking associated with the more conventional SLA process resulting in a printing time in the order of minutes with DLP rather than minutes to hours with SLA to build a 1 cm^3^ construct (Liang et al., 2021; Levato et al., 2023). Nevertheless, the overall printing time is also largely influenced by the selected sample height and the interplay between the reactivity of the proposed bioresin formulation and the applied optimized light dose per layer (Liang et al., 2021). Moreover, the positive lateral resolution attained with vat polymerization methods relies heavily on the optical voxel size (SLA: laser spot size, DLP: LCD/DMD pixel size), reactivity of the bioresin, degree of light dispersion resulting from the applied cell density, light wavelength and light dose distribution in and around the voxel of interest (Liang et al., 2021; Levato et al., 2014; Li W. et al., 2023). Practically, for both DLP and SLA, this translates into a positive lateral bioprinting resolution of several tens of micrometers (Zandrini et al., 2023). It should, however, be taken into account for DLP that a trade-off exists between the projection area and the pixel size since decreasing the pixel size for an enhanced resolution also results in a reduced projection area due to the inherent build-up of the LCD/DMD light projection system (Li W. et al., 2023). Furthermore, the axial resolution is determined by the movement resolution of the build platform together with the light penetration depth which is inversely correlated with the molar extinction coefficient and concentration of the photo-initiator, the amount of photo-absorber or -inhibitor added to the bioresin and the bioresin viscosity (Ng et al., 2020; Goodarzi Hosseinabadi et al., 2022; Li W. et al., 2023). The addition of photo-absorbers or -inhibitors allows to control the light penetration, delaying the onset of photo-polymerization and hereby improving the resolution through alleviating the mismatch between the light penetration depth and the selected printing layer thickness which should be slightly smaller than the light penetration depth to allow adherence between the different printing layers (Liang et al., 2021; Goodarzi Hosseinabadi et al., 2022; Li W. et al., 2023). The viscosity of the bioresin can also be altered whereby an increase in the density of the bioresin causes a reduction in light penetration depth together with decreasing the risk of encapsulated cell sedimentation (Goodarzi Hosseinabadi et al., 2022; Levato et al., 2023). However, care should be taken that the bioresin viscosity remains beneath a threshold of 10 Pa.s in order to allow it to flow back between printing of two subsequent layers (Ng et al., 2020). The hereby associated risk of encapsulated cell sedimentation can also be prevented through the selection of appropriate photo-crosslinkable moieties within the curable modified polymer in the bioresin that allow for an adequately fast crosslinking rate (Levato et al., 2023).

For example, SF isolated from *B. Mori* cocoons with a methacrylation degree of 67.3% (SFMA) has been used in various concentrations (10–15 – 25 w/v%) in combination with 2 million mouse calvarial pre-osteoblast (MC3T3-E1) cells/mL hereby enabling DLP-based bioprinting of grid-like constructs encapsulating 0.1 million cells (Rajput et al., 2022). The visco-elastic bioinks exhibited compressive moduli ranging from 12 kPa for the 10 w/v% network up to 41 and 96 kPa for the 15 and 25 w/v% networks respectively. The mass loss after 21 days as a measure of the degradation rate comprised 91%, 65% and 49% respectively for the 10, 15 and 25 w/v% network. Interestingly, network stiffening was observed (nevertheless only measured for the 15 w/v% network) over the time course of the degradation due to the SF β-sheet formation in the presence of water leading to a temporal crystallinity increase of the network. The 15 w/v% SFMA-network incorporated MC3T3-E1 cells showing the highest cell area, cell perimeter, aspect ratio and lowest circularity. This aligns well with the findings from Huebsch et al. and Chaudhuri et al. who showed that the force response of the cell strain is dependent on the initial matrix stiffness. This response then determines whether the cytoskeleton-associated adhesion complexes can be assembled (not the case in very compliant substrates), if the cells can generate enough force to deform the network (not the case in very rigid substrates) and ultimately whether matrix reorganization and cell spreading can take place (Huebsch et al., 2010; Chaudhuri et al., 2016). The complex interplay between network degradation and stiffening in this case then further aids the encapsulated elongated cells to deposit their own ECM and further enhance late-stage osteogenic differentiation through the presence of calcium deposits as was confirmed for the 15 w/v% network in culture medium both with and without osteogenic supplements (Caliari and Burdick, 2016; Loebel et al., 2019; Li X. et al., 2023).

**FIGURE 6:**
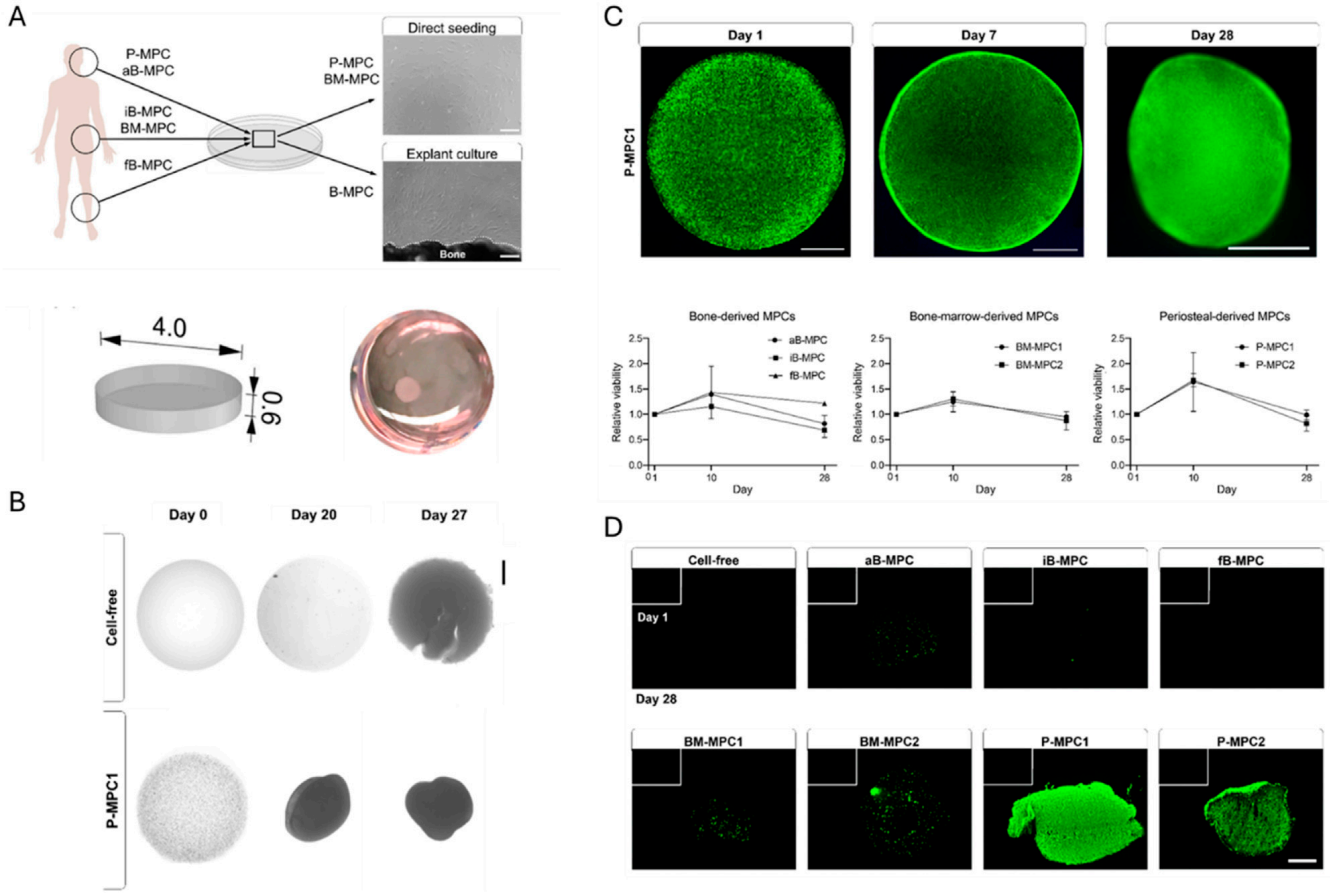
The isolation of mesenchymal progenitor cells (MPC) from alveolar bone (aB), fibula bone (fB), iliac crest bone (iB), iliac crest bone marrow (BM) and periosteum of the mastoid (P) for encapsulation within bioprinted constructs (scale bars: 200 μm) **(A).** Cell-mediated construct shrinkage after 27 days (scale bar: 1,000 µm) **(B).** Cell viability and metabolic activity within **(C)** and mineralization of **(D)** the produced constructs (scale bar in **(C)** 1,000 μm, scale bar in **(D)** 500 µm). Reproduced from Amler et al. (2021) under an open access license.

The importance of the used cell type was illustrated by Amler et al. who encapsulated various mesenchymal progenitor cells (from alveolar bone (aBSC), fibula bone (fBSC), iliac crest bone (iBSC), iliac crest bone marrow (iBMSC) and periosteum of the mastoid (PMSC)) in 8 w% GelMA networks (DS not specified) through SLA bioprinting (0.1 w% LAP) at a density of 20 million cells/mL (Figure 6A) (Amler et al., 2021). The identification of the most suitable cell type to be used for bioprinting is important in order to obtain a cell type capable of efficiently undergoing osteogenesis with a fast and easy expansion that is obtained with straightforward and minimally invasive harvesting causing low morbidity. Most of the cell types used in that study were obtained through bone or periosteum explantation of the zone of interest. Only the iBMSCs were harvested through fine needle aspiration. Furthermore, in the case of iBMSC and PMSC, two donors were included to take into account the donor variability. The bone-derived mesenchymal progenitor cells were expanded through explant outgrowth, the cells obtained from the bone marrow were directly seeded for multiplication and the periosteal progenitors were seeded after tissue digestion.

Bioprinted construct shrinkage was observed over 28 days due to cellular contraction which can have a major influence on the outcome of the biofabricated construct due to a reduced nutrient delivery since included features like bioprinted channels might be partially blocked due to cellular bridging (Figure 6B) (Soliman et al., 2022). The extent of contraction and its influence are highly depending on the cell type(s) used, the cellular concentration, the design of the construct and the photo-crosslinkable network applied. Nevertheless, in this case, highly viable cellular constructs were obtained (Figure 6C). By day 10, the highest metabolic activity over the 28-day period across all investigated cell types was observed which was correlated to an enhancement of extracellular matrix secretion in the second week causing impaired diffusion of the metabolic activity dye at later time points. Moreover, upon differentiation, the encapsulated cells lost their highly proliferative status hereby clarifying the diminishing metabolic activity trend in the third and fourth week of bioprinted construct cultivation. Gene expression level quantification over the 28-day period and visualization of the amount of calcification after 4 weeks in the constructs allowed for comparing the differentiation level of the mesenchymal progenitor cells from different sources (Figure 6D). The aBSCs were the superior bone-derived progenitor cell type in terms of osteogenic differentiation when compared to cells from fibular or iliac crest bone. Nevertheless, these aBSCs still appeared to be at an early differentiation stage after 28 days with early markers RUNX2, ALPL and COL1A1 being significantly upregulated after 4 weeks, no downregulation of the later *s*ecreted protein acidic and cysteine rich (SPARC, encoding osteonectin) marker and only deposition of nodule-like mineralization structures. IBMSCs also showed only deposition of nodule-like mineralization structures but nevertheless already downregulated the SPARC marker as a sign of higher maturity. In contrast, PMSCs showed a high and uniform mineralization signal in combination with downregulated early marker genes, a downregulated SPARC gene and a stable mature secreted phosphoprotein 1 (SPP1, encoding OPN) gene rendering them a clinically relevant cell type for further bioprinting studies given their high proliferation capacity and the fact that they can be obtained in a minimally invasive way. Nevertheless, donor variability should also be taken into account. Here, it was found that IBMSCs show a higher variability compared to PMSCs. However, more extensive research is needed to fully capture the bioprinting outcome of progenitor cells from more sources and different donors.

Natural polymers are ideally suited for cell encapsulation with long-term survival yet, are limited in attaining high-resolution bioprinting with sufficient construct shape fidelity (Levato et al., 2021). Therefore, Lim et al. added 1 wt% GelMA (DS 60%) to 10 wt% methacrylated poly (vinylalcohol) (PVAMA) in combination with 0.2 mM/2 mM Ru/SPS (tris-bipyridylruthenium (II) hexahydrate/sodium persulfate) photo-initiator, 1 wt% Ponceau 4R photo-absorber and 5 million human BMSCs/mL for DLP-based bioprinting of highly defined cell-interactive constructs (Lim et al., 2018). The addition of GelMA resulted in similar physicochemical properties, positive and negative resolutions down to 50 µm yet resulted in a significantly higher compressive modulus as compared to pure 10 wt% PVAMA. Also, supplementation of the modified gelatin allowed enhanced long-term encapsulated cell survival up to 14 days and a qualitatively higher ALP production after 7 days thanks to the fact that the bioprinted stem cells were able to sense the surrounding network resulting from the cell-interactive groups present in the gelatin backbone.

**FIGURE 7:**
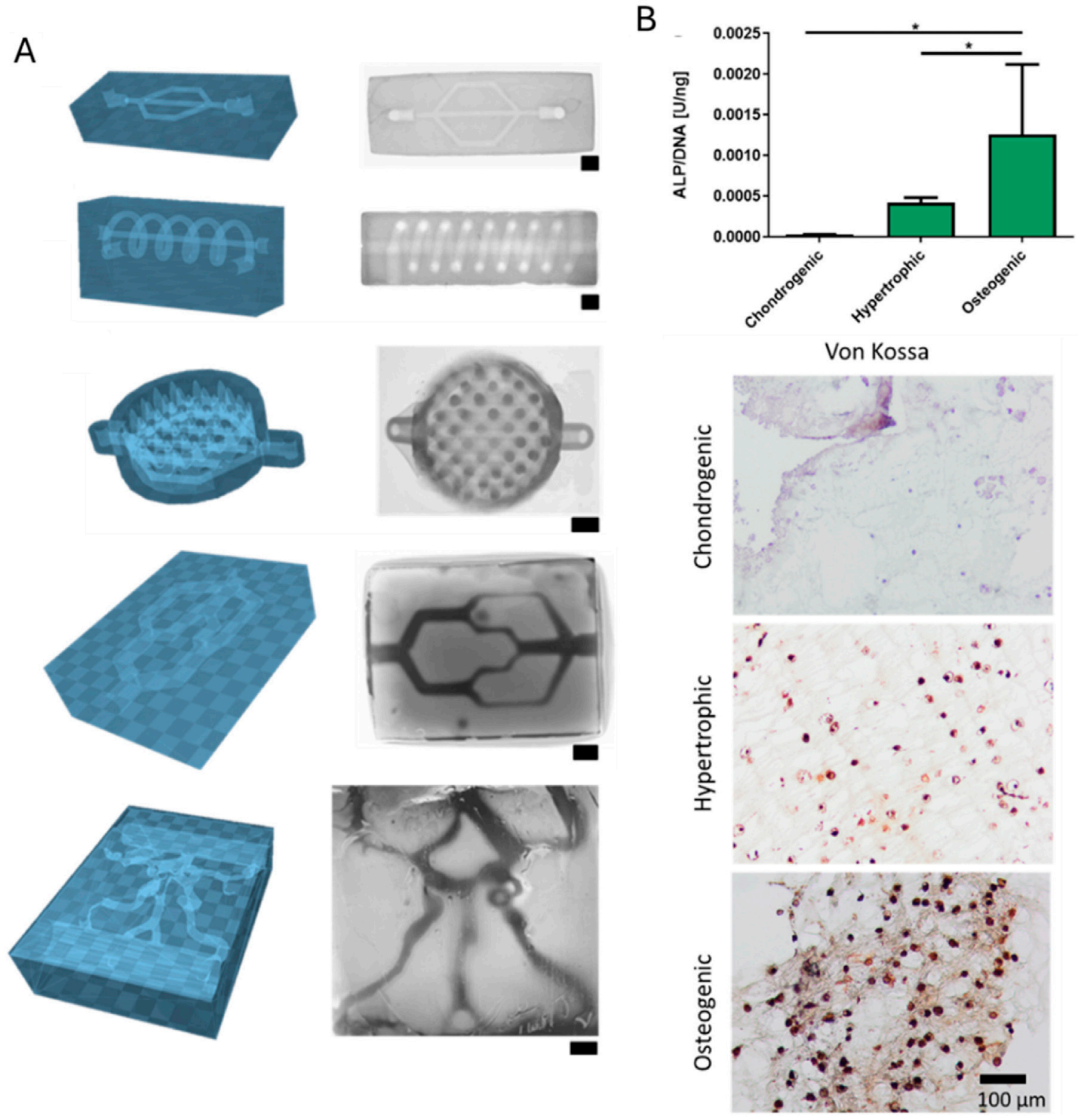
DLP-printing of LTS-GelMA (ichthyic gelatin modified with methacryloyl groups) hydrogels allowing the production of complex, perfusable networks imaged through stereomicroscopy (from top to bottom): a branched microfluidic chip, a horizontal channel with a spiraling tube around it, mimicry of intestinal epithelium crypt-villi with an open, branched channel network underneath, a branched microfluidic network with varying channel diameter and mimicry of a portion of the blood vessels within the convoluted, irregular vessel-like human Willis circuit (scale bars: 1 mm) **(A)**. Alkaline phosphatase activity and Von Kossa mineralization staining of encapsulated bone marrow-derived stem cells in DLP-bioprinted LTS-GelMA constructs subjected to chondrogenic, hypertrophic and osteogenic media (scale bar: 100 µm) **(B)**. Reproduced from Levato et al. (2021) under an open access license.

By increasing the natural polymer content, Levato et al. succeeded in bioprinting highly defined complex cold water fish gelatin-based constructs exhibiting complex channels with a perfusable lumen (diameter <200 µm) (Figure 7A) (Levato et al., 2021). The lower hydroxyproline content in gelatin from ichthyic origin resulted in lower melting point triple helices with thermal stability at room temperature and decreased mechanical properties when compared to other types of gelatin (from porcine or bovine sources) making it a suitable candidate for the low-viscosity biofabrication technique DLP. Low-temperature soluble (LTS) bioresins consisted of either a methacryloyl- (DS 90%, LTS-GelMA) or a norbornene- (DS 85%, LTS-GelNB crosslinked with PEG4SH) modified gelatin in combination with the aforementioned photo-initiator and -absorber. The effect of the step-versus chain-growth crosslinking mechanism is nicely illustrated upon determining the resolution where the 10 w/v% LTS-GelMA resin showed the best approximation of the 50 µm positive resolution (non-significant difference with LTS-GelNB). This was in contrast to the 5 w/v% LTS-GelNB network significantly outperforming the LTS-GelMA resin in reaching a closer CAD-CAM mimicry of 100 µm negative resolution despite the comparable compressive moduli, penetration depth and critical energy. Nevertheless, the step-growth ink’s crosslinkability decreased after 30 min likely due to loss of reactivity because of thiol-persulfate redox reactions even in the absence of light hereby limiting the production of larger structures extending in the vertical direction. Therefore, bioprinting was only considered for the 10 w/v% LTS-GelMA resin encapsulating 10 million equine BMSCs/mL which were able to undergo osteogenic differentiation. A higher alkaline phosphatase activity and more extensive calcium deposition could be observed when the encapsulated cells were exposed to osteogenic medium as compared to hyperthrophic or chondrogenic media underlining the importance of the supplemented biochemical cues on the final outcome of the construct (Figure 7B).

**FIGURE 8:**
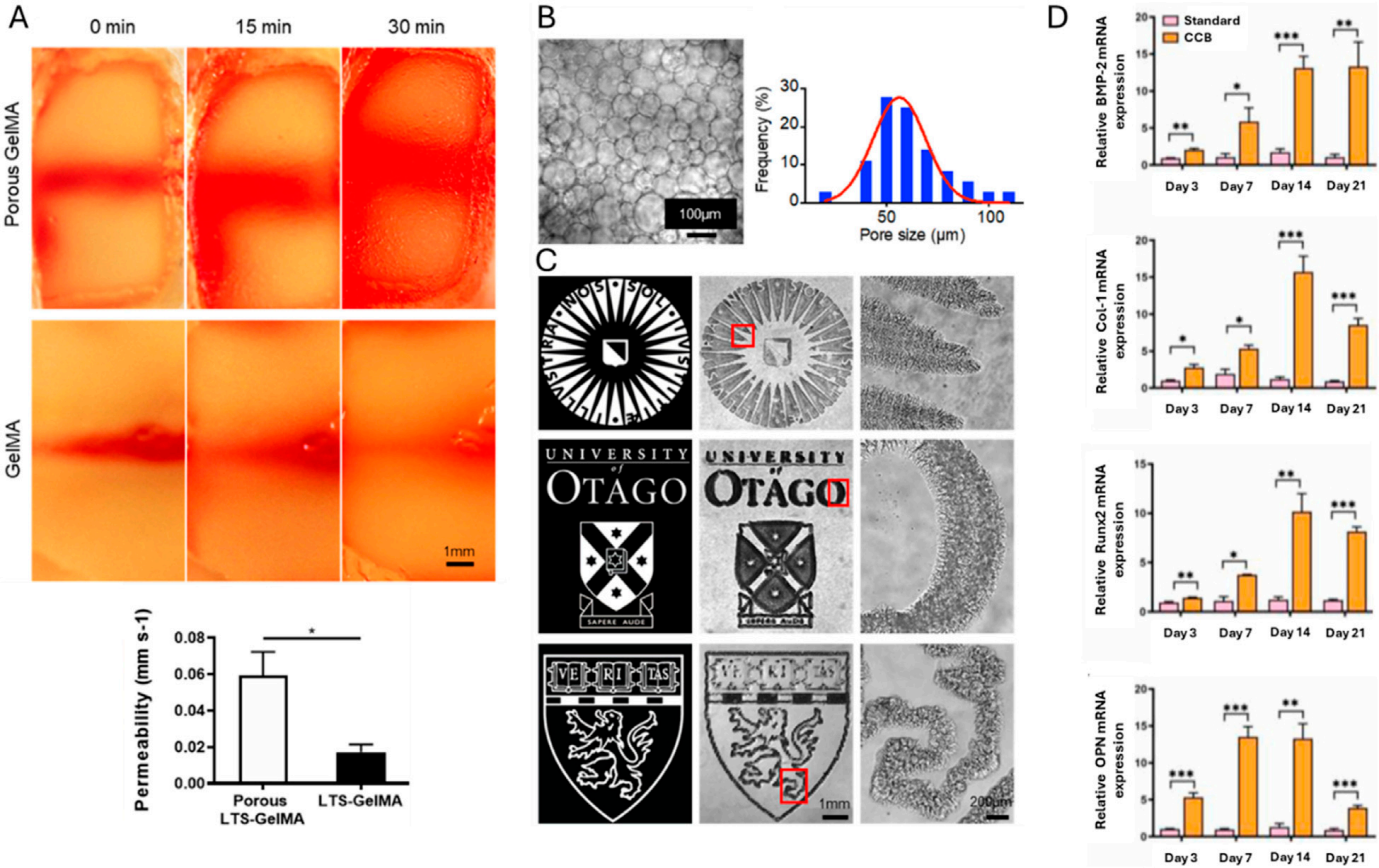
Enhancing DLP-printed construct permeability **(A)** through the use of a second porogen droplet phase (1.6 w/v% poly (ethylene glycol)) in crosslinked methacryloyl-modified ichthyic gelatin **(B)** while still enabling high-resolution prints **(C)** (scale bar in (A) 1 mm, scale bar in **(B)** 100 µm and scale bars in **(C)** 1 mm and 200 µm). Higher osteogenic gene expression in the rat DPSC concentrated porogen bioink (CCB) as compared to single rat DPSC encapsulated controls without porogen phase **(D)**. Reproduced from Levato et al. (2021), Zhu et al. (2025) under an open access license.

Levato et al. further also successfully investigated the use of a second porogen phase (1.6 w/v% PEG with a molar mass of 300 kDa) to create an emulsion bioresin which is immiscible with the combined LTS-GelMA resin (15 w/v%) as a means of enhancing the permeability towards nutrients and metabolic waste products through porogen removal after incubation in a hydrated environment (Figures 8A–C) (Levato et al., 2021). This void-forming behavior was further evaluated through the addition of 3.33 w/v% dextran (molar mass of 500 kDa) to 10 w/v% GelMA (DS not specified) by Tao et al (Tao et al., 2022). Constructs with and without dextran were then DLP-bioprinted in combination with rat bone MSCs (concentration not specified). The void-forming constructs exhibited a significantly decreased compressive modulus, faster degradation and an enhanced diffusion leading to an enhanced proliferation over a 5-day period, an increased migration over 10 days and higher cellular spreading at day 7. The increased permeability also resulted in an enhanced YAP nuclear expression in contrast to the control where the lower YAP signal mainly remained in the cytoplasm. This resulted in significant upregulation of the early RUNX2 and ALP markers on day 7 and day 14 followed by a significant increase in the late OSX marker after 2 weeks. The observed osteogenesis might have arisen from both the enhanced nutrient and metabolic waste product diffusion of the highly metabolically active stem cells as well as the increased ability of the encapsulated cells to deposit their own matrix (Caliari and Burdick, 2016; Loebel et al., 2019; Li X. et al., 2023). They could even show that 8 weeks *in vivo* implantation of the DLP-bioprinted constructs in a cranial defect in Sprague-Dawley rats gave rise to a more gradually calcified bone integrated within the host bone. Interestingly, when rat DPSCs (concentration not specified) were incorporated into the void-forming phase (3.33 w/v% 500 kDa dextran), the *in-situ* birth of stem cell spheroids could be observed in the remaining 10 w/v% GelMA (DS not specified) matrix (Zhu et al., 2025). These spheroids showed enhanced proliferation, *in vitro* osteogenic differentiation (Figure 8D) and *in vivo* endodontic tissue regeneration capability as compared to rDPSC-encapsulating 10 w/v% GelMA controls without a porogen phase.

### 3.2 Two-photon-based bioprinting

Two-photon lithography (TPL) is a laser-scanning technique that relies on the non-linear bridging of the excited state energy gap through simultaneous absorption of two photons (Ovsianikov et al., 2012; Greant et al., 2023). The probability of two-photon absorption scales with the square of the incident light intensity and is inversely proportional to the fourth power of the distance from the laser focal plane (Ovsianikov et al., 2012; Lay et al., 2020). Hence, by adjusting the laser power, this effect can be exploited in a highly localized volume in the focal spot (<1 μm^3^) to allow light-based photo-crosslinking, -grafting, -degradation or -ablation (Ovsianikov et al., 2012; Greant et al., 2023; Levato et al., 2023). Overall, the outcome of TPL in terms of printability, printing time, attainable sample size, resolution, shape fidelity and print stability is mainly affected by the constituents of the bioresin (photo-crosslinkable polymer concentration and reactivity, cell type and concentration, photo-initiator concentration and efficiency), the optical set-up, sample post-processing and the delivered light dose to the resin (Ovsianikov et al., 2012; Lay et al., 2020; Greant et al., 2023; Levato et al., 2023).

Given the highly localized focal volume, TPL achieves subdiffraction minimum feature sizes within the order of 10^–7^ m (Ovsianikov et al., 2014; Lay et al., 2020; Greant et al., 2023). Given that for raster-scanning techniques, speed scales with volume, this results, together with the reported resolution, in a printing time in the range of hours to create a 1 cm^3^ bioprinted construct (Levato et al., 2023). Nevertheless, an increase in the number of lasers or light beams has already been applied to augment the writing speed while still enabling high resolution (Levato et al., 2023). In order to avoid overheating (except in situations where photo-ablation is desired) with the high intensity femtosecond lasers, the photo-reactivity of the applied bioresins should be high in combination with a high transparency at the used wavelength hereby circumventing linear absorption and/or irradiation blockage (Ovsianikov et al., 2012; Lay et al., 2020). Next to this, the viscosity should be adequate (>10 Pa.s) to prevent cellular sedimentation as well as to avoid structure deformation during the printing process (Lay et al., 2020; Levato et al., 2023).

**FIGURE 9:**
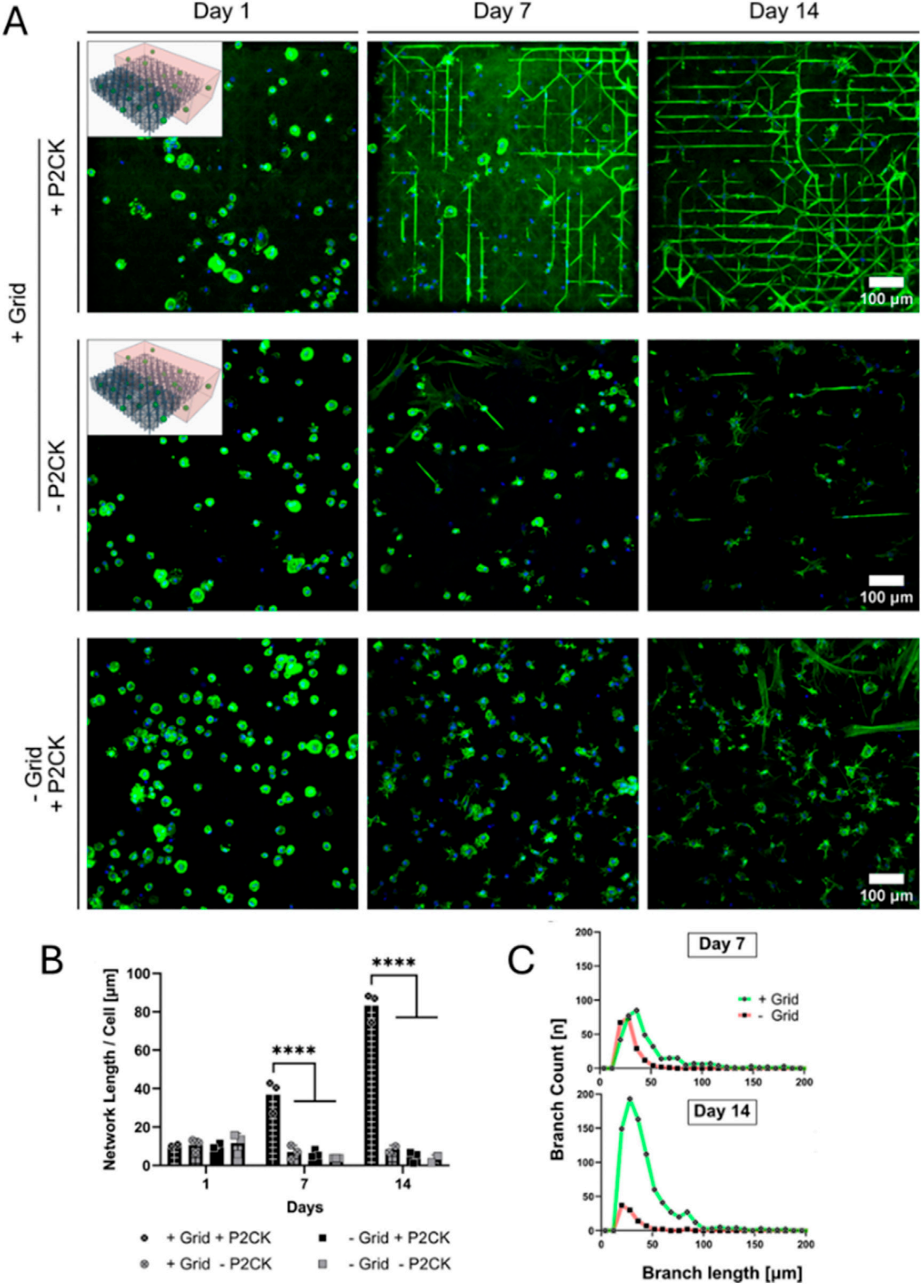
P2CK photosensitization and laser ablation synergistically affect 3D human mesenchymal stem cellular network formation (scale bar: 100 µm) **(A)** in terms of normalized network length **(B)** and branch count **(C)**. Reproduced from Gehre et al. (2024) under an open access license.

Two-photon ablation (TPA) has been used to create an interconnected cell network (1 µm diameter) hereby mimicking the native, late-stage osteocyte lacunar-canalicular microarchitecture (Gehre et al., 2024). In order to enhance the ablation efficiency and to create a human BMSC-compatible ablation energy dose (100 J/cm^2^), a two-photon photo-sensitizer (0.5 mM sodium 3,3′-((((1E,1′E)-(2-oxocyclopentane-1,3-diylidene)-bis(methaneylylidene))-bis(4,1-phenylene))-bis(methyl-azanediyl))-dipropionate, P2CK) was added to photo-crosslinked, cell-encapsulated GelMA networks (5 w/v% GelMA with DS of 56%, 0.05% LAP, 2.5 million cells/mL, 365 nm, 3 J/cm^2^). The 3D ablated networks were successfully colonized by day 7 with long protrusions exceeding 40 µm and the establishment of cellular functional contacts through gap junctions (Figure 9). Moreover, it was shown that the embedded cells preferentially used the confining channels over the ability to spread through proteolytic remodeling within the constraining GelMA network. This also affected the ALP activity after 7 and 14 days with a slightly higher ALP activity for patterned networks versus the non-ablated control. This study nicely illustrates that approaches for stimulating encapsulated cell spreading for enhanced osteogenesis are not limited to the permissive character of the applied bioresin but are also heavily influenced by the presented topography on the cell level.

### 3.3 Volumetric bioprinting

Computed axial lithography (CAL), tomographic volumetric printing (VP) or volumetric additive manufacturing (VAM) where light energy is delivered to a 3D volume instead of a point (e.g. SLA, TPL) or a plane (e.g. DLP), allows to overcome the limited throughput and the constrained geometric capabilities evoking the need for support or sacrificial materials associated with more conventional layer-by-layer biofabrication approaches (Shusteff et al., 2017; Kelly et al., 2019; Loterie et al., 2020; Bernal et al., 2022; Thijssen et al., 2023; Jing et al., 2024). The accumulated 3D dose distribution on a resin container whose rotation is time sequenced with the light projection, results from the superposition of 2D cross-sectional intensity-modulated image projections from multiple angles hereby allowing to locally reach the solidification threshold of the resin according to the specified input design model of the desired object (Kelly et al., 2019; Loterie et al., 2020). Overall, the outcome of tomographic volumetric bioprinting in terms of printability, printing time, attainable sample size, resolution, shape fidelity and print stability is mainly affected by the constituents of the bioresin (photo-crosslinkable polymer concentration and reactivity, cell type and concentration, concentration and efficiency of photo-initiators (and -inhibitors)), the light projection optics and computation, the delivered light dose to the resin and the post-processing method applied (Shusteff et al., 2017; Bernal et al., 2019; Bernal et al., 2022; Kelly et al., 2019; Loterie et al., 2020; Rizzo et al., 2021; Madrid-Wolff et al., 2023; Thijssen et al., 2023; Jing et al., 2024).

The ability to construct prints volume-wise allows several orders of magnitude faster printing speeds - compared to layer-by-layer biofabrication techniques – requiring a printing time in the order of seconds to build a 1 cm^3^ construct allowing improved scalability and enhanced encapsulated cellular viability and functionality (Bernal et al., 2019; Kelly et al., 2019; Rizzo et al., 2021; Levato et al., 2023). The resolution of volumetric constructs depends on the viscosity and the reactivity of the resin, potential presence of scattering elements in the resin, the pixel size and the magnification of the light modulating projection system, the spatial coherence of the light source and the tomographic dose reconstruction accuracy (Bernal et al., 2019; Kelly et al., 2019; Loterie et al., 2020; Madrid-Wolff et al., 2023). The positive resolution of the technique is limited to >40 µm whereas the lowest reported negative resolution achieved with VAM is around 100 µm (Rizzo et al., 2021; Bernal et al., 2022; Cianciosi et al., 2023; Madrid-Wolff et al., 2023). The components of the volumetric photoresin should be optimized to have a high reactivity but low absorbance, allowing for moderate light attenuation so that the solidification threshold can be reached across the entire build volume (Kelly et al., 2019; Rizzo et al., 2021; Thijssen et al., 2023). Furthermore, scattering in volumetric resins either polymerization-, dispersed resin additive- (encapsulated cells, spheroids or organoids) or embedded macroscale object-induced, should be minimized through the use of refractive index adjusting agents, by adjusting the optical set-up or through incorporating this effect within the computational reconstruction (Bernal et al., 2022; Madrid-Wolff et al., 2022; Thijssen et al., 2023). Moreover, sedimentation should be limited through either dose optimization algorithms or the modulation of the resin viscosity which should surpass a value of 10 Pa.s (Loterie et al., 2020; Rizzo et al., 2021; Thijssen et al., 2023). Finally, after exposure, care should be taken that, given the short printing times, low conversion of the printed construct might heavily impact the print stability requesting the need for post-curing (or crystallization-inducing processes) (Thijssen et al., 2023).

**FIGURE 10:**
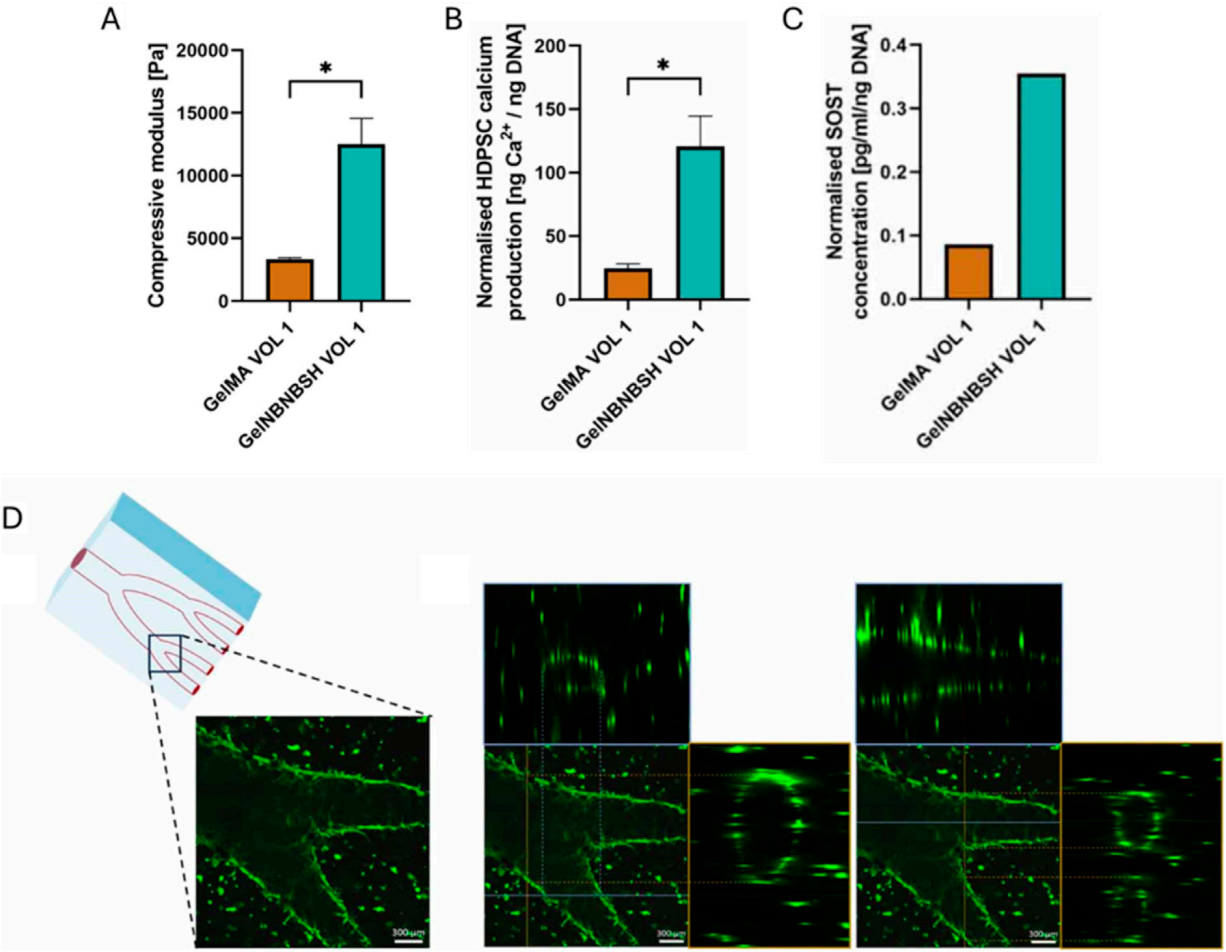
Influence of shifting the crosslinking density and chemistry from chain-to step-growth on the mechanical properties **(A)**, the calcium production of bioprinted HDPSCs within these matrices at day 21 with respect to their DNA content **(B),** sclerostin signaling of bioprinted HDPSCs within these matrices at day 21 with respect to their DNA content **(C)**. Perfusable step-growth crosslinked, volumetric bioprinted constructs could be produced with highly viable encapsulated HDPSCs after 21 days (subfigures with a blue and orange frame represent intersection images indicated by a blue and orange line in the original image, scale bars: 300 µm) **(D)**. Reproduced from Duquesne et al. (2025) with permission.

Gehlen et al. successfully exploited volumetric bioprinting to print vascularized constructs targeting osteogenesis by encapsulating 3 million human BMSCs/mL within a 5% GelMA (DS 57%) perfusable construct (Gehlen et al., 2023). 5% GelMA was chosen since an enhanced osteogenic differentiation was observed as reflected by the increased relative gene expression of the osteocyte marker gene podoplanin (PDPN) compared to denser 10% GelMA networks. Despite the low mechanical properties associated with the 5% network, this network might have allowed for enhanced diffusion of nutrients and waste products together with a more active spreading of the encapsulated cells while depositing their own pericellular matrix which has also been found to be a determining factor in osteogenesis (Caliari and Burdick, 2016; Loebel et al., 2019; Li X. et al., 2023). Further decreasing the concentration with or without the addition of unmodified gelatin was not considered since this could limit the handleability of the volumetrically printed construct. Next, the effect of cellular crosstalk in an encapsulated co-culture of endothelial (0.6 million human umbilical vein endothelial cells (HUVECs)/mL) and stem cells (3 million human BMSCs/mL) was evaluated on osteogenic gene expression in comparison to encapsulated stem cells on their own. The authors observed significantly upscaled early osteogenic markers for the monoculture whereas, for the co-culture, significantly increased osteoblastic markers, an enhanced ALP gene expression and activity and higher early osteocytic markers were seen. However, up to 6 weeks, no calcium deposits were observed through micro-CT in both mono- and co-cultures together with the absence of the mature osteocytic marker sclerostin (SOST) which was correlated to the need for enhanced maturation. Duquesne et al. applied in this regard a stiffer 5 w/v% GelNBNB/GelSH (DS 176/72) matrix as compared to 5 w/v% GelMA (DS 95) (Figure 10A) as volumetric printing bioink (Duquesne et al., 2025). Encapsulated human DPSCs (1 million cells/mL) exhibited enhanced late-stage osteogenic differentiation markers (mineralization, Figure 10B and SOST-expression; Figure 10C) when encapsulated within perfusable step-growth crosslinked, volumetric bioprinted constructs (Figure 10D).

## 4 Conclusions, current limitations and future opportunities

**FIGURE 11:**
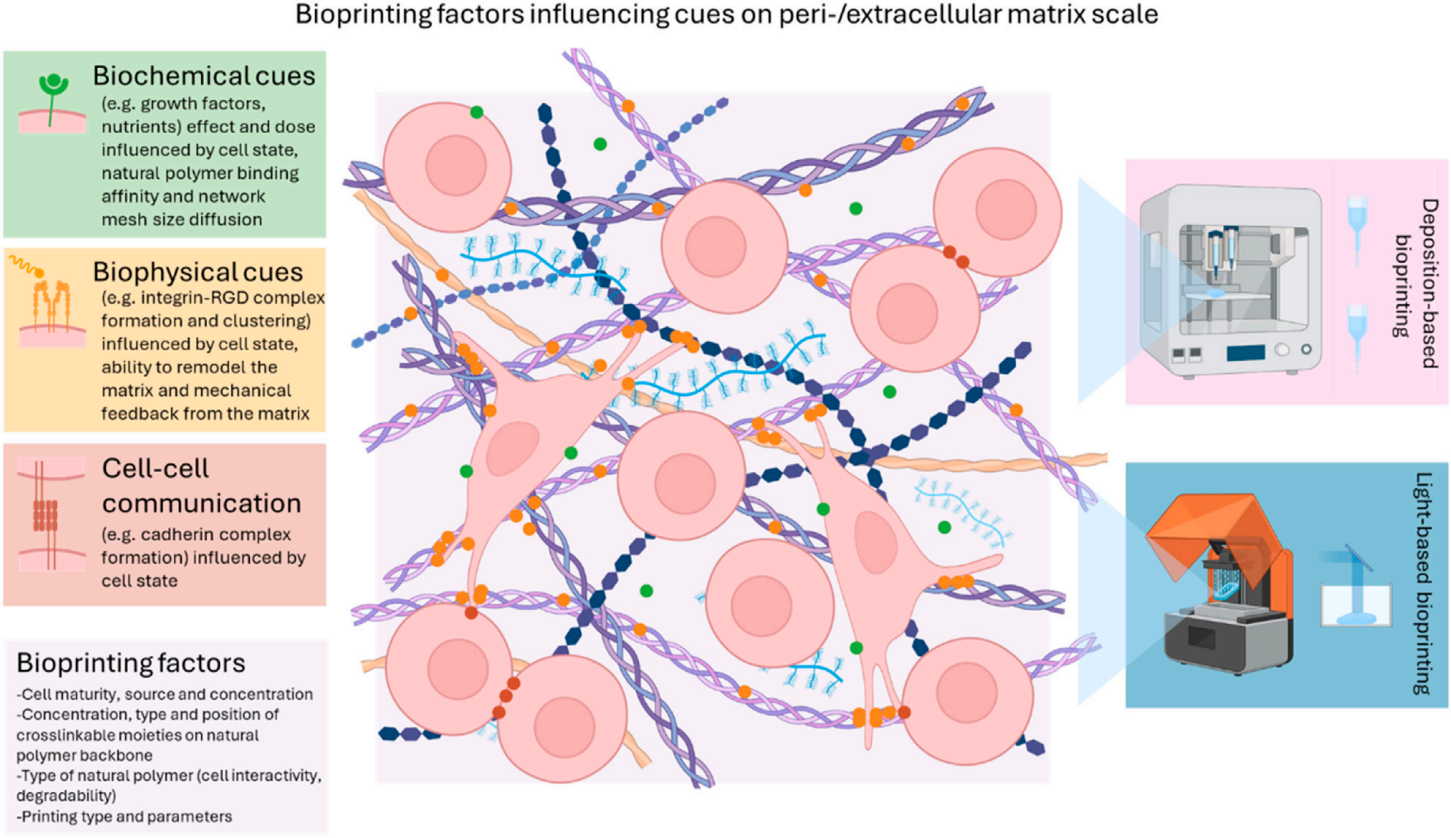
Overview of how bioprinting factors affect encapsulated cell-cell communication, biochemical and biophysical cues at the peri-/extracellular matrix niche. The intention of this summarizing figure is not to fully replicate the highly complex ECM environment but to provide an overview of the mechanisms discussed in this review. Figure created with BioRender.

The osteoregenerative outcome of a bioprinted construct highly depends on the presented cues from the encapsulating matrix (concentration, type and location of crosslinkable moieties on the natural polymer backbone together with the type of selected natural polymer affecting cellular interactivity and degradability), the used printing strategy (type and parameters) and the maturity, source and concentration of the utilized cell type (Figure 11). When selecting all the bioprinting factors to ensure optimal osteogenesis, the surrounding matrix should be designed in such a way that it is a mechanically performant network capable of inducing osteogenesis while still being permissive for the cell to cluster cell-interactive ligands and hence perceive the presented network. In this regard, given the variability of reported mechanical properties inductive for osteogenesis characterized through various techniques, there is a need to look further into a workflow that allows to visualize and characterize better the cell-interactive ligand availability and clustering within the 3D network. Additionally, the reported bioinks are often designed in the first place to result in an adequate printability rather than to result in a maximal osteoregenerative capacity. In this way more promising bioink formulations might be missed. There is also a need to develop more step-growth crosslinking bioinks given the listed advantages towards cellular encapsulation. However, for thiol-ene systems, bioink stability still remains a massive hurdle towards their widespread translation whereas the use of Schiff base and enzymatic crosslinking bioinks is limited due to their limited spatiotemporal control (Echalier et al., 2019; Van Hoorick et al., 2019; Levato et al., 2021). Moreover, the local dose of biochemical cues should be tightly controlled to ensure maximum efficiency and a desired, safe outcome. Furthermore, biophysical and biochemical cues might be overshadowed by extensive cell-cell communication which is an often-overlooked cue that can nevertheless heavily influence osteogenesis. Finally, in order to optimally test the presented peri-/extracellular matrix environment, clinically relevant cell types should be selected that are obtained through straightforward and minimally invasive harvesting and which allow for a fast and easy expansion (Moroni et al., 2018b).

In order to fully elucidate the predominant effects at play in these complex systems targeting osteogenesis, standardization in the reporting of the cues as well as the *in vitro/in vivo* outcome are required (Moroni et al., 2018a). Material properties such as e.g. substrate elasticity should therefore be evaluated using the same broadly applied technique and parameters. Biological expressions such as e.g. calcium production should be normalized with respect to the DNA content to differentiate the effect of osteogenesis from the effect of proliferation. Moreover, quality assurance evaluation should be performed on the components of the bioink so that consistent and reproducible results can be attained.

The printing type and parameters also highly affect the presented cues towards the encapsulated cells in the bioprinted construct targeting osteogenesis. An overview of the discussed printing techniques including their viscosity requirements, cell density limits, as well as the minimum feature widths, throughput and some *in vitro* challenges is summarized in Table 4. In general, light-based bioprinting techniques allow a higher spatial resolution compared to deposition-based bioprinting technologies therefore resulting in a superior mimicry of the tissue micro-environment structural complexity such as complex vascularization trees and innervation networks within bone spanning different length scales (Lim et al., 2018). On the other hand, deposition-based bioprinting techniques in general allow multi-material processing to produce more heterogeneous constructs therefore providing a superior mimicry of the tissue micro-environment biological complexity hereby carefully bridging the mechanical, cell source and compositional requirements for osteo-, angio- and neurogenesis (Levato et al., 2021). Nevertheless, to enable functional and scalable hierarchical constructs serving osteogenesis and incorporating vascularization and innervation, multiple materials and/or techniques should ideally be combined to tackle key challenges in order to exploit the full (Mandrycky et al., 2016; Moroni et al., 2018b; Kunwar et al., 2019; Castilho et al., 2020; Größbacher et al., 2023; Ribezzi et al., 2023; Rizzo et al., 2023) clinical potential of the field:

**TABLE 4:** Summary of bioink requirements (i.e. viscosity, maximum cell density, ability to print spheroids), potential output (i.e. minimum feature width, throughput) and *in vitro* challenges of the discussed bioprinting techniques.

Bioprinting techniques	Viscosity [mPa.s]	Maximum cell density [cells/mL]	Ability to print spheroids	Minimum feature width [µm]	Printing time of 1 cm^3^	*In vitro* challenges	References
Extrusion	30–6.10^7^	10^8^	Yes	200–1,000	Minutes to hours	- Mechanical stresses during printing reducing cell viability - Contact between nozzle and construct increasing risk of construct distortion and contamination	Chang et al. (2008), 2011; Malda et al. (2013), Murphy and Atala (2014), Hölzl et al. (2016), Diamantides et al. (2019), Ning et al. (2020), De Moor et al. (2021), Xu et al. (2022a), Levato et al. (2023)
Inkjet	3.5–12	10^6^	No	10–50	Minutes to hours	- Mechanical and/or thermal stresses during printing and upon deposition reducing cell viability - Sedimentation of cells due to low viscosity requirement	Liu et al. (1970), Murphy and Atala (2014), Hölzl et al. (2016), Mandrycky et al. (2016), Madou, 2018; Xu et al. (2019), 2022a; Li et al. (2020)
SLA	250–10^4^	10^7^	Yes	10	Minutes to hours	Sedimentation of cells due to low viscosity requirement	Soman et al. (2013), Loterie et al. (2020), Schwab et al. (2020), Grigoryan et al. (2021), Levato et al. (2023), Zandrini et al. (2023)
2PP	>10^4^	10^7^	Yes	0.1	Hours	High photo-reactivity of resins to avoid overheating	Nano Scribe (2025), Ovsianikov et al. (2012), 2014; Nguyen and Narayan (2017), Lay et al. (2020), Dobos et al. (2021), Valente et al. (2022), Levato et al. (2023), Cantoni et al. (2024)
DLP	250–10^4^	10^8^	No	10	Minutes	Sedimentation of cells due to low viscosity requirement	Li et al. (2019b), Loterie et al. (2020), Mao et al. (2020), Schwab et al. (2020), Levato et al. (2023), You et al. (2023), Zandrini et al. (2023)
VP	>10^4^	10^7^	Yes	40	Seconds	- Light scattering due to encapsulated cells, spheroids or particles - Post-printing processes are required to increase print stability (e.g. post-curing, crystallization-inducing processes)	Bernal et al. (2019), 2022; Loterie et al. (2020), Madrid-Wolff et al. (2022), Thijssen et al. (2023)

### 4.1 Mechanical performance

The tissue engineered construct should allow mechanical stability after implantation at the defect site up until the moment the newly formed bone can gradually take over this role (Preethi Soundarya et al., 2018). Hence, to reduce fibrous tissue formation and stimulate callus bridging, mechanical discontinuities should be prevented at the scaffold-bone interface (Prasadh and Wong, 2018). However, large variations are observed in specific target mechanical values since these highly depend on the anatomical defect and its different loadings, in addition to age, gender and possible co-morbidities of the patient (Velasco et al., 2015). Nevertheless, when comparing the order of magnitude of target values (e.g. Young’s modulus: 10^7^–10^10^ Pa) versus mechanical properties reported for photo-crosslinked natural polymeric hydrogels (e.g. Young’s modulus: 10^3^–10^5^ Pa), it becomes clear that reinforcement strategies are paramount towards further clinical translation (Velasco et al., 2015; Cidonio et al., 2019a; Alcala-Orozco et al., 2020; Wang J. et al., 2024).

Following a biomimetic strategy, natural polymer-based hydrogels have in this regard been combined with a ceramic phase. The concentration, distribution, size, aspect ratio, charge and chemistry of this reinforcing phase determine whether natural hydrogel crosslinking is maintained and/or whether additional (physical and/or chemical) bonds are being created. Based on this multi-factorial outcome, the mechanical properties are altered. A more than two-fold increase in elastic modulus was observed by Yu et al. through the addition of xonotlite (5 wt%) to GelMA (10 w/v%, degree of substitution (DS) not specified) thanks to the presence of attractive forces between the polymer network and the nano-fillers (Yu et al., 2024). The same strengthening effect was observed upon addition of nano beta-tricalcium phosphate (0, 1, 3 or 5 w/v%) to GelMA (5 w/v%, DS not specified) and alginate (1 w/v%) by Zhang et al. as long as the scattering ceramic fraction was not too high to decrease the crosslinking degree (Zhang et al., 2024). The latter effect on the crosslinking degree was also reported by Sun et al. when graphene oxide nanosheets (1 mg/mL) were incorporated within photo-crosslinked gelatin- and silk-based networks (5 w/v% GelMA, 3 w/v% SFMA and 5 w/v% GelDA DS not specified) hereby effectively decreasing the mechanical properties (Sun X. et al., 2023). By first mixing calcium phosphate nanoparticles with gelatin and subsequent methacrylation (final concentrations and DS not specified), Bhattacharyya et al. succeeded in creating a more controlled size, aspect ratio and distribution of the particles leading to improved mechanical properties as compared to conventional methods which involve first the methacrylation of gelatin followed by nanoparticle mixing (Bhattacharyya et al., 2022). Choi et al. reported on a silane modification of silica nanoparticles (10 wt%) exhibiting strong repulsive forces preventing aggregation and allowing good dispersibility and an improved Young’s modulus when introduced within GelMA networks (15 wt%, DS not specified) (Choi et al., 2021). When increasing the whitlockite/hydroxyapatite nanoparticle ratio (25%–100%) within gelatin- and alginate-based networks (7 w/v% GelMA DS 81%, 4 w/v% alginate and 0.5 w/v% gelatin), Ghahri et al. observed a decreased compressive modulus due to the repulsion of the negatively charged surface of whitlockite with the carboxylic acid groups of the natural polymer network decreasing the chemical and ionic crosslinking degree (Ghahri et al., 2023). Finally, Zhu et al. reported on the covalent attachment of bioactive glass particles (concentration not specified) to a gelatin- and alginate-based network (final concentrations of gelatin and oxidized alginate (oxidation degree 30%) not specified) as an effective means to increase the compressive modulus (Zhu et al., 2022).

**FIGURE 12:**
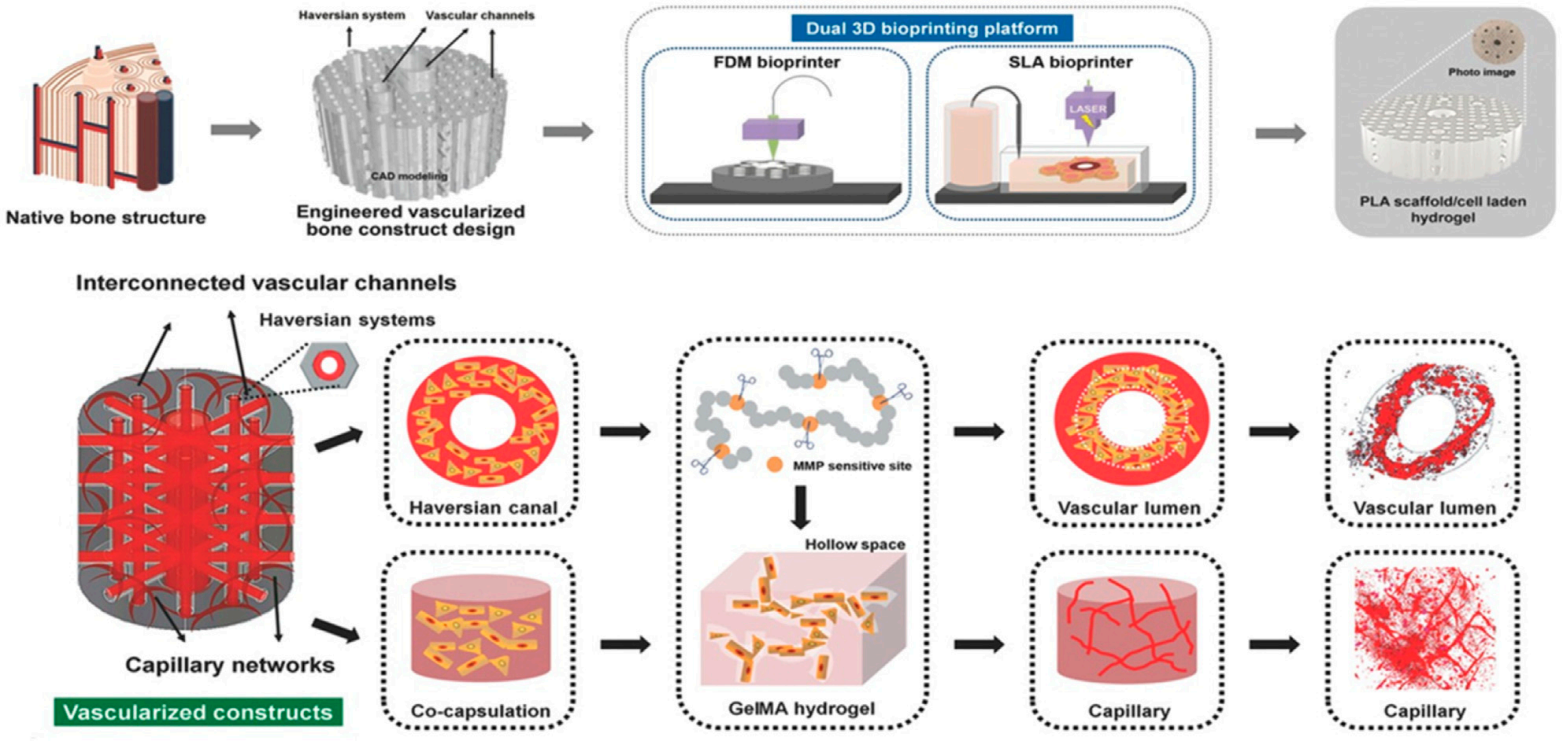
Multi-technique strategy (FDM + SLA) to achieve a perfusable, mechanically performant construct with interconnected vascular channels and capillary networks. Reproduced from Cui et al. (2016) with permission.

Despite promising results, the maximum Young’s modulus obtained for bioprinted ceramic/photo-crosslinkable natural polymer composites is situated around 10^6^ Pa which is not sufficient considering the target value range (*vide supra*) (Liu et al., 2024). Therefore, more emphasis has to be placed on multi-material and/or multi-technique strategies that allow the combination of a mechanically performant macroscopic system with adequate cellular niches for optimal stimulation of bone healing. Cui et al. combined in this regard fused deposition modeling/fused filament fabrication (FDM/FFF) of poly (lactic acid) with SLA of GelMA (10 wt%, DS not specified) to achieve perfusable tissue engineered constructs with a Young’s modulus around 10^8^ Pa (Figure 12) (Cui et al., 2016). Moreover, multi-material extrusion-based scaffolds of magnesium-reinforced (20 wt%) poly (ɛ-caprolactone) and poly (lactic-co-glycolic acid) were combined with strontium-reinforced (1.5 μg/mL) GelMA (5 wt%, DS 60%) and GelMA (concentration and DS not specified) respectively to finally achieve a Young’s modulus around 10^7^ Pa (Alcala-Orozco et al., 2022; Rodrigues et al., 2024). All reported mechanical properties are nevertheless heavily depending on the specific printing design and hence further research is needed to elegantly combine and spatiotemporally balance the mechanical reinforcement fraction, the bioprinted part and adequate porosity allowing for tissue ingrowth. Moreover, mechanical testing parameters should be more standardized to allow better comparison.

### 4.2 Immunological response

**FIGURE 13:**
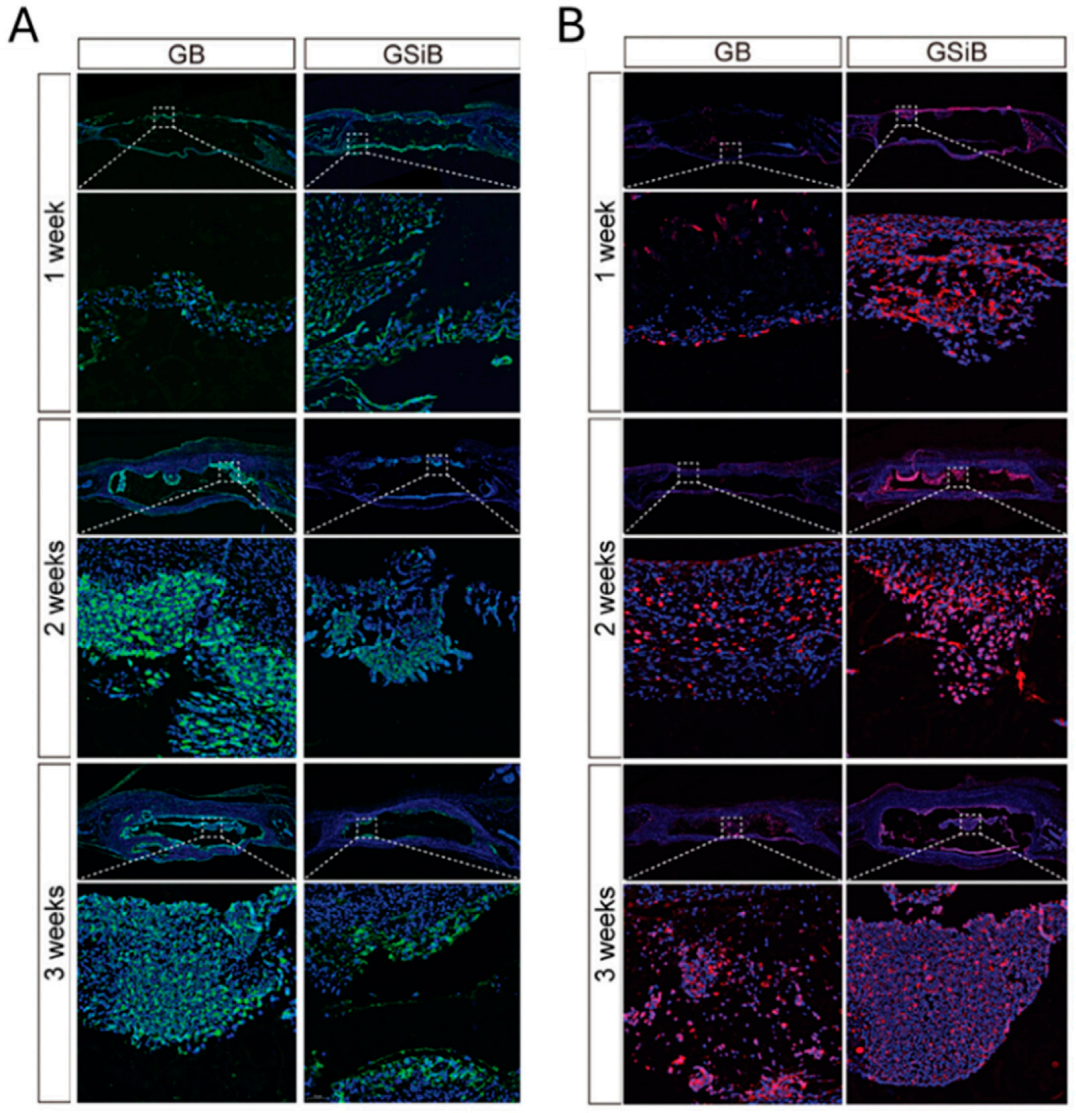
Immunofluorescent marker staining related to M1 **(A)** and M2 **(B)** polarization around the implantation area of GB (10% GelMA (DS not specified) + 1 million rat BMSCs/mL) and GSiB (10% GelMA +0.5 mg/mL silicon-substituted calcium phosphate +1 million rat BMSCs/mL). Reproduced from Liu et al. (2024) with permission.

Before neurovascularized bone ingrowth can occur, immunological signaling will largely determine the tissue response (Du et al., 2024). Chronic pro-inflammatory (M1 polarization) signaling will lead to a tissue repair impediment and fibrosis development whereas immunomodulation to a pro-regenerative (M2 polarization) environment after hours to days enables to initiate optimal bone healing (Du et al., 2024; Duquesne et al., 2025). Despite its role as the tissue engineering gold standard, the use of GelMA within bioinks for extrusion-based, volumetric or DLP-based bioprinting resulted in the expression of M1-associated markers both *in vitro* and *in vivo* between 7 and 21 days (Du et al., 2024; Liu et al., 2024; Yu et al., 2024; Duquesne et al., 2025). Interestingly, shifting to step-growth crosslinking chemistry gave rise to overall lower levels of pro-inflammatory cytokines at later time points underlining the need to validate more bioinks relying on step-growth photo-crosslinking chemistry (Duquesne et al., 2025). Moreover, the addition of manganese and strontium to GelMA-based bioinks allowed immunomodulation towards an M2 type and the subsequent secretion of cytokines related to tissue regeneration, hereby effectively stimulating osteogenic differentiation *in vitro* and bone healing *in vivo* (Figure 13) (Du et al., 2024; Liu et al., 2024; Yu et al., 2024). Yet, more studies are needed to further understand the immunomodulatory role towards bone healing and to implement this knowledge in biomaterial design.

### 4.3 Vascularization and innervation

Nerves and blood vessels play important roles in bone development, homeostasis and regeneration (Hankenson et al., 2011; Marenzana and Arnett, 2013; Li Z. et al., 2019; 2025). During bone regeneration, the fracture will be firstly innervated which is a crucial step in the formation of the ossification center (Li Z. et al., 2019). The nerves will release neurotransmitters, neuropeptides, neurotrophins regulating the bone regenerating micro-environment (Sun et al., 2020; Sun et al., 2023 W.). Next, the bone defect will be vascularized allowing the provision of nutrients, oxygen and growth factors and the removal of waste products as well as the recruitment of osteoprogenitor cells (Hankenson et al., 2011; Marenzana and Arnett, 2013; Sun W. et al., 2023). Despite the fact that delayed or absent vascularization and innervation result in impaired fracture healing, hydrogel-based approaches targeting neuro-vascularized bone regeneration are lacking (Hankenson et al., 2011; Li Z. et al., 2019; Meyers et al., 2020). The different cells involved in bone formation, innervation and vascularization require different micro-environments for optimal proliferation and differentiation evoking the need of a scaffold heterogenous in biophysical and biochemical properties (Wan et al., 2020; Li et al., 2025). However, the developed bone scaffolds to date are often lacking this multi-tissue focus.

**FIGURE 14:**
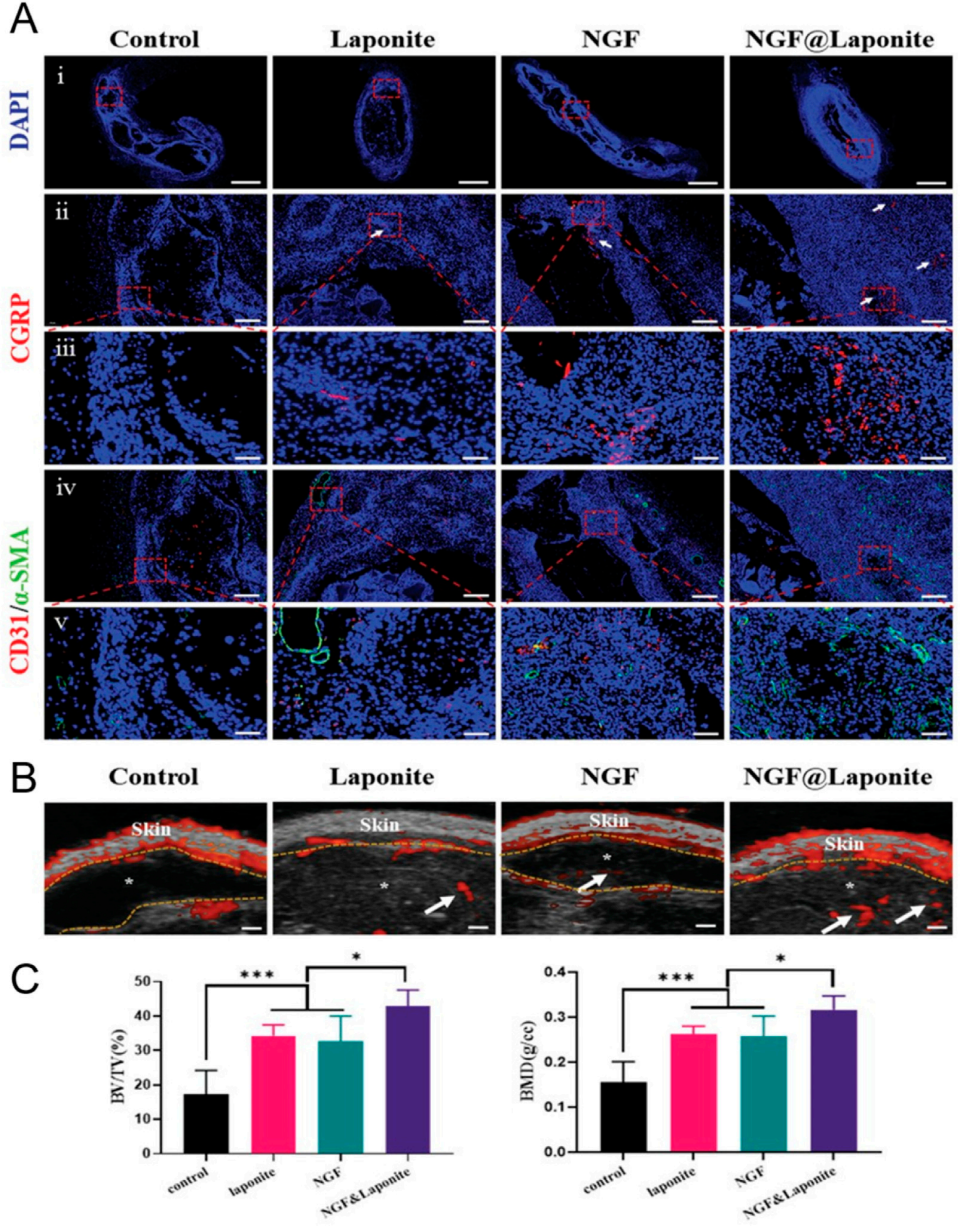
Bioprinted gelatin-methacryloyl/alginate methacrylate (GelMA/AlgMA) constructs encapsulating human bone marrow derived stem cells (BMSCs). The evaluated groups are GelMA/AlgMA (control), GelMA/AlgMA with Laponite (laponite), GelMA/AlgMA with nerve growth factor (NGF) and GelMA/AlgMA with NGF-loaded Laponite (NGF&Laponite). **(A)** Immunofluorescence staining of calcitonin gene-related peptide (CGRP), cluster of differentiation 31 (CD-31) and alpha-smooth muscle actin (α-SMA) after 14 days of subcutaneous implantation. Scale bars: i) 1,000 µm ii) 200 μm, iii) 50 μm, iv) 200 µm and v) 50 µm. **(B)** Ultrasound images after 14 days of subcutaneous implantation with hydrogel and vasculature indicated with asterisks and white arrows respectively. Scale bars: 1,000 µm. **(C)** Bone-to-tissue volume ratio (BV/TV) [%] and bone mineral density (BMD) [g/cc] obtained through µ-CT after 8 weeks of implantation in the cranial defect. Reproduced from Li et al. (2022) with permission.

Fortunately, bioprinting facilitates the fabrication of multi-material and hetero-cellular scaffolds with complex architecture and heterogenous biophysical/biochemical properties targeting multiple tissue type regeneration (Li Q. et al., 2023). Below, representative examples targeting neuro-vascularized bone regeneration through bioprinting will be reviewed. In the first set of examples, solely stem cells were selected. Li et al. encapsulated Laponite loaded with nerve growth factor (NGF, 20 mg/mL) and rat BMSCs (10 million cells/mL) within a mixture of GelMA (5%, DS not specified) and alginate methacrylate (AlgMA, 2%, DS not specified) (Li et al., 2022). The reversible binding of NGF with Laponite evokes a slower release of the growth factor. Subcutaneous implantation of bioprinted constructs revealed an improved osteogenic differentiation through calcitonin gene-related peptide (CGRP) release of sensory neurons stimulated by Laponite and NGF. Additionally, the most pronounced innervation and vascularization were detected using immunofluorescence (based on CGRP, cluster of differentiation 31 (CD-31) and alpha-smooth muscle actin (α-SMA), day 14) and ultrasound imaging (day 14) in the experimental group (Figures 14A,B). Finally, the positive effect on bone regeneration was validated in a cranial defect model after 8 weeks of implantation using µ-CT, hematoxylin and eosin staining, and Masson’s trichrome staining (Figure 14C). Using a similar strategy, mesoporous silica nanoparticles were loaded with propranolol (PRN) and CGRP, causing the sustained release of PRN, CGRP and Si ions and thus an improved osteogenesis and angiogenesis within the bioprinted construct (Guo and He, 2023). Both studies prove that loading growth factors or therapeutic agents into nanoparticles represents a promising approach to achieve (more) controlled release. Another system in which osteogenesis was elegantly combined with angio- and neurogenesis exploited DLP-printing of 10 w/v% GelMA (DS not specified) to encapsulate 50 million human dental pulp-derived stem cells/mL microspheroids (Qian et al., 2023). The researchers showed that compared to 2D cell seeding onto 10 w/v% photo-crosslinked GelMA sheets and tissue culture plate, the 3D microspheroids showed equivalent osteogenic (odontogenic) differentiation (through dentin matrix acidic phosphoprotein 1 (DMP1) and dentin sialophosphoprotein (DSPP) expression) but significantly higher angio- (through VEGFα and angiopoietin 1 (ANGPT1) expression) and neurogenesis (through growth associated protein 43 (GAP43) and microtubule associated protein 2 (MAP2) expression) underlining the importance of dimensionality and cellular concentration influencing the biological outcome of a printed construct.

**FIGURE 15:**
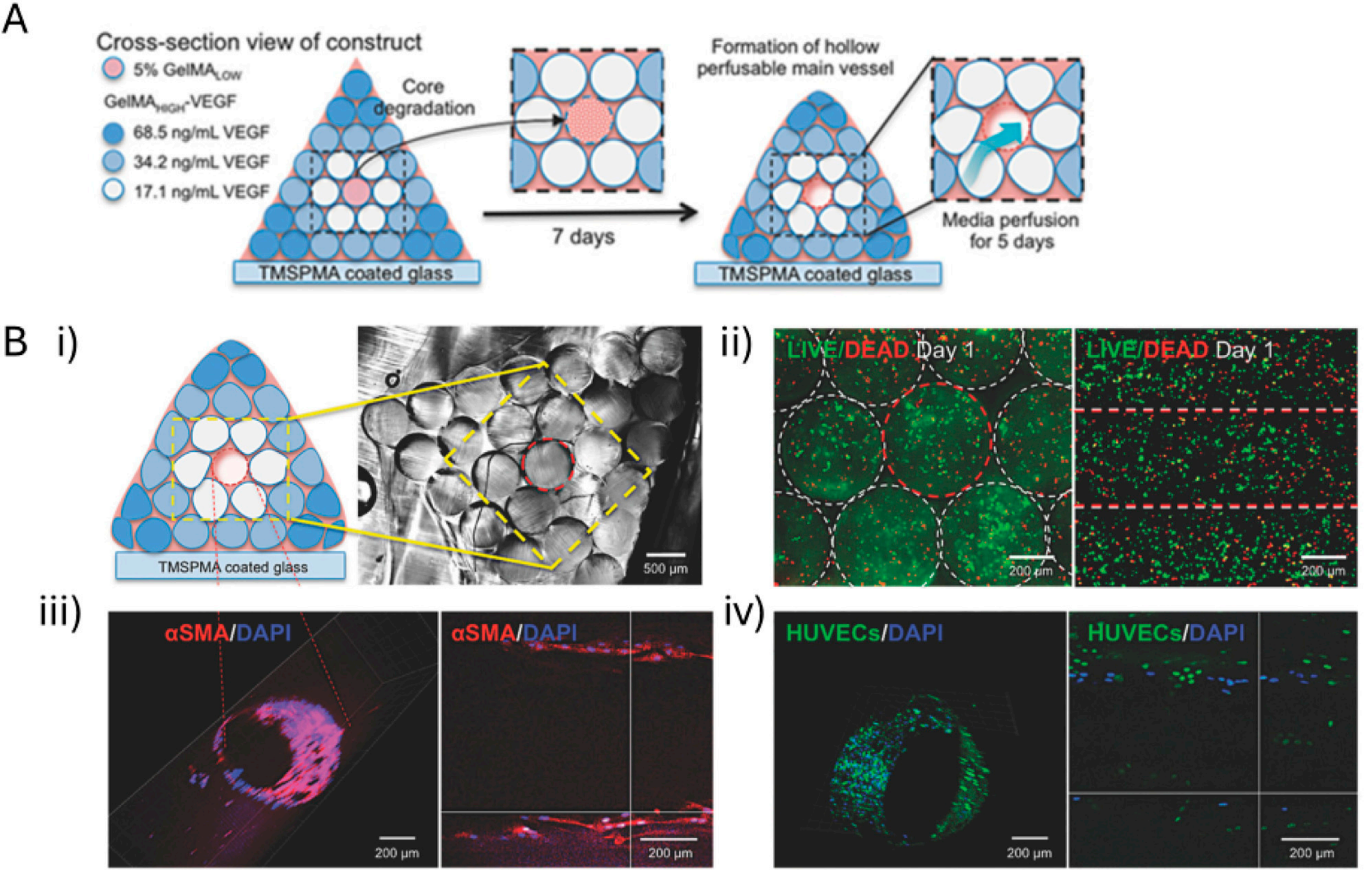
Extrusion bioprinting of gelatin-methacryloyl (GelMA) bioinks containing different covalently linked vascular endothelial growth factor (VEGF) concentrations (17.1–34.2–68.5 ng/mL) to obtain a VEGF gradient for introducing vascularization. **(A)** Schematic overview of the bioprinting design whereby the center consists of rapidly degradable GelMA (5 w/v% with DS 34%) resulting in a hollow feature over time serving as a perfusable channel within the construct centered within a VEGF dose gradient network. **(B)** i) Cross-section of the bioprinted construct. ii) Live/dead staining of the encapsulated green fluorescence protein-labeled human umbilical vein endothelial cells (GFP-HUVECs) and human bone marrow-derived stem cells (hBMSCs). iii) Immunofluorescence staining of alpha smooth muscle actin (α-SMA) visualizing the differentiated hBMSCs at day 12 post-culture. Nuclei are stained blue (DAPI). iv) Confocal images of GFP-HUVECs lining the hollow channel. Nuclei are stained blue (DAPI). Reproduced from Byambaa et al. (2017) with permission.

Besides using solely mesenchymal stem cells, also neural cells and/or endothelial cells were encapsulated to replicate better the cellular composition of the various tissues present within bone. Firstly, the beneficial effect of mesenchymal stem cell–neural cell co-culture in treating bone defects was illustrated by Zhang et al. who extrusion bioprinted sequentially two GelMA (DS not specified, 6 w/v%) bioinks supplemented with calcium silicate (CS, 2%) nanowires encapsulating either rat BMSCs (2 million cells/mL) or Schwann cells (2 million cells/mL) (Zhang et al., 2022). After 4 and 8 weeks implantation of extrusion bioprinted constructs in a cranial defect, the experimental group revealed the most pronounced stimulation of osteogenesis (based on µ-CT and immunofluorescence staining of OCN and OPN) and neurogenesis (based on immunofluorescence staining of CGRP and neurofilament). This stimulation was attributed to the synergistic effect of the CS nanowires releasing bioactive ions including Ca and Si ions, and the neural-bone cell co-culture. Since Schwann cells regulate the proliferation and osteogenic differentiation of stem cells via the release of exosomes, an alternative bioink encapsulated BMSCs and Schwann cells’ exosomes (Jones et al., 2019; Wang T. et al., 2023). In addition to the beneficial effect on osteogenesis and neurogenesis, subcutaneous implantation of the extrusion bioprinted constructs revealed after 14 days a robust blood flow inside the constructs based on ultrasound imaging (Wang T. et al., 2023). Secondly, combining mesenchymal stem cells and endothelial cells in one construct is a promising strategy when targeting vascularization. The endothelial cells can be either exploited to generate large vessels through lining of engineered, hollow features or to generate microvasculature. Those hollow structures have been generated through inclusion of non-covalently crosslinked biomaterials or rapidly degradable covalently crosslinked hydrogels. Shen et al. extrusion bioprinted a porous GelMA scaffold (5 wt%, 5 million bone MSCs/mL) of which the pores were initially filled with PLA-PEG-PLA (10 wt%) encapsulating rat aortic endothelial cells (RAOECs, 5 million cells/mL) (Shen et al., 2022). Within a short time frame (∼1 h), PLA-PEG-PLA was dissolved and resulted in an improved seeding efficiency of endothelial cells compared to conventional seeding. Both *in vitro* and *in vivo* experiments revealed an improved effect of both the experimental seeding approach and the co-culture on the formation of new bone and vascularization. Endothelial cells lining the inside of an engineered vessel can also be achieved via multi-axial bioprinting of GelMA bioinks containing specific cells stimulating angiogenesis or osteogenesis as the outer shells, and gelatin as the inner shell (Zhang et al., 2024). Zhu et al. reported enhanced vascularization connected to the host vasculature thanks to co-culture spheroids consisting of HUVECs and human DPSCs (concentration not specified) formed after the introduction of a void-forming phase (3.33 w/v% 500 kDa dextran) in GelMA (10 w/v%, DS not specified) (Zhu et al., 2025). A final alternative strategy entails the inclusion of rapidly degradable GelMA in the inner core of the construct. Byambaa et al. extrusion bioprinted the construct’s inner core of rapidly degradable VEGF-conjugated GelMA (5 w/v%, DS 34%) encapsulating HUVECs and hBMSCs, and an outer core of GelMA (10 w/v%, DS 94%) grafted with a gradient VEGF concentration encapsulating hBMSCs and nano silicate particles (Figure 15A) (Byambaa et al., 2017). The co-culture of MSCs and HUVECs lined the created channel, with the MSCs differentiating into smooth muscle cells, which accelerates the formation and maturation of a vascular network (Figure 15B). Both VEGF and the co-culture positively influenced vasculogenesis by stimulating capillary network formation and endothelial cell spreading. MSCs encapsulated in the outer region differentiated towards osteoblasts due to the presence of encapsulated silicate nanoparticles and VEGF. In this example, the half-life of the growth factor was enhanced by covalently attaching it to the gelatin backbone. Next to grafting, also the small molecule drug fingolimod (1,000 ng/mL) can be used to obtain longer half-life in order to stimulate migration, proliferation and capillary-like structure formation of endothelial cells and thus stimulate angiogenesis (similar to 100 ng/mL VEGF) (Yang et al., 2022). In a final example, multiple 3D bioprinting platforms were used (FFF of poly (lactic acid) and SLA of 10 wt% GelMA (DS not specified)) in which human MSCs were first seeded on the poly (lactic acid) scaffold followed by SLA bioprinting of a co-culture of human MSCs in combination with HUVECs (Cui et al., 2016). The successful combination of FFF with SLA allowed to mimic bone at different hierarchical scales thereby showing the ability to spatially control bioactive factor arrangement, cellular organization and mechanical loading. The use of dynamic perfusion of the construct in combination with the presentation of biochemical cues (growth factors BMP-2 and VEGF) highly stimulated both osteogenesis (in terms of ALP activity, collagen type I synthesis and mineralization) and angiogenesis (VEGF expression).

Interestingly, when rat DPSCs (concentration not specified) were incorporated into the void-forming phase (3.33 w/v% 500 kDa dextran), the *in-situ* birth of stem cell spheroids could be observed in the remaining 10 w/v% GelMA (DS not specified) matrix (Zhu et al., 2025). These spheroids showed enhanced proliferation, *in vitro* osteogenic differentiation and *in vivo* endodontic tissue regeneration capability as compared to rDPSC-encapsulating 10 w/v% GelMA controls without a porogen phase.

To conclude, while constructs composed of photo-crosslinkable natural polymers utilizing single bioprinting technologies offer significant advantages in generating an appropriate osteoid niche to allow osteogenic differentiation, the current focus should be extended towards constructs combining multiple material and/or multiple printing techniques. This multifaceted approach is essential to achieve functional and scalable constructs enabling *in vivo* bone regeneration. Such constructs would not only support osseous tissue formation but also vascularization and innervation, as well as meet macroscopic mechanical target values. Additionally, given the advantages of step-growth crosslinkable bioinks for cell encapsulation, including their promising immunomodulatory properties, a paradigm shift from conventional chain-growth crosslinkable bioinks to step-growth crosslinkable bioinks is of paramount importance. Although excellent papers have been published addressing various elements of this complex problem, current constructs fail to meet all necessary requirements.
